# Genome-Wide Association Study Reveals Novel Genomic Regions for Grain Yield and Yield-Related Traits in Drought-Stressed Synthetic Hexaploid Wheat

**DOI:** 10.3390/ijms19103011

**Published:** 2018-10-02

**Authors:** Madhav Bhatta, Alexey Morgounov, Vikas Belamkar, P. Stephen Baenziger

**Affiliations:** 1 Department of Agronomy and Horticulture, University of Nebraska-Lincoln, Lincoln, NE 68583, USA; madhav.bhatta@huskers.unl.edu (M.B.); vikas.belamkar@unl.edu (V.B.); 2 International Maize and Wheat Improvement Center (CIMMYT), 06511 Emek, Ankara, Turkey; a.morgounov@cgiar.org

**Keywords:** marker–trait association, haplotype block, genes, root traits, D-genome, genotyping-by-sequencing, single nucleotide polymorphism, durum wheat, bread wheat, complex traits

## Abstract

Synthetic hexaploid wheat (SHW; 2*n* = 6*x* = 42, AABBDD, *Triticum aestivum* L.) is produced from an interspecific cross between durum wheat (2*n* = 4*x* = 28, AABB, *T. turgidum* L.) and goat grass (2*n* = 2*x* = 14, DD, *Aegilops tauschii* Coss.) and is reported to have significant novel alleles-controlling biotic and abiotic stresses resistance. A genome-wide association study (GWAS) was conducted to unravel these loci [marker–trait associations (MTAs)] using 35,648 genotyping-by-sequencing-derived single nucleotide polymorphisms in 123 SHWs. We identified 90 novel MTAs (45, 11, and 34 on the A, B, and D genomes, respectively) and haplotype blocks associated with grain yield and yield-related traits including root traits under drought stress. The phenotypic variance explained by the MTAs ranged from 1.1% to 32.3%. Most of the MTAs (120 out of 194) identified were found in genes, and of these 45 MTAs were in genes annotated as having a potential role in drought stress. This result provides further evidence for the reliability of MTAs identified. The large number of MTAs (53) identified especially on the D-genome demonstrate the potential of SHWs for elucidating the genetic architecture of complex traits and provide an opportunity for further improvement of wheat under rapidly changing climatic conditions.

## 1. Introduction

Drought is one of the most important abiotic stresses that reduce crop productivity and is expected to increase with the change in climate [1]. Erratic rainfall patterns caused by climate change may aggravate drought stress and will have a major impact on agriculture [2,3]. The most prominent example of the impact of drought stress on agriculture was the 2012 drought stress in the United States, where moderate to extreme drought stress occurred across the central agricultural states that resulted in crop harvest failure for corn (*Zea mays* L.), sorghum (*Sorghum bicolor* L.), and soybean (*Glycine max* L.), and the agriculture loss due to drought was estimated to be $30 billion [4]. To cope with the challenges of drought stress, plant breeders have been focusing on improving drought tolerance since several decades [2,3,5]. However, the drought tolerance is a complex phenomenon as most of the traits associated with drought tolerance are polygenic in nature, and understating the genetic architecture of drought tolerance is still underway [3] including in wheat (*Triticum sps*.) [2]. Wheat is one of the most important staple cereal crops mainly grown under rainfed conditions [3,6] and is expected to suffer from drought stress [3]. Therefore, breeding for drought tolerance and identifying genomic regions and underlying candidate genes associated with drought tolerance are important for wheat improvement.

Bread wheat (*T. aestivum* L.) has limited genetic and phenotypic diversity available for breeding for drought tolerance [2]. This is mainly due to the genetic bottleneck experienced during its origin and subsequent domestication [7,8]. Diversity can be increased through the production of synthetic hexaploid wheat (SHW) and its utilization in breeding programs [9,10,11]. Synthetic hexaploid wheat (2*n* = 6*x* = 42, AABBDD) is produced from an interspecific cross between durum wheat (2*n* = 4*x* = 28, AABB, *T. turgidum* L.) and goat grass (2*n* = 2*x* = 14, DD, *Aegilops tauschii* Coss.). The SHWs are reported to have significant genetic variation for biotic [12,13] and abiotic stresses resistance [2,10,14]. However, previous studies focused mainly on biotic stresses including leaf rust (incited by *Puccinia triticina*) [13,15,16], stem rust (incited by *P. graminis*) [15,16], stripe rust (incited by *P. striiformis*) [12,15,16], Fusarium head blight (incited by *Fusarium graminearum*) [13], yellow spot (incited by *Pyrenophora tritici-repentis*) [15,16], septoria nodorum (incited by *Parastagonospora nodorum*) [15,16], Septoria tritici blotch (incited by *Mycosphaerella graminicola*) [13,15], cereal cyst nematode (incited by *Heterodera avenae*) [15], crown rot (incited by *F. pseudograminearum*) [16], and root-lesion nematode (incited by *Pratylenchus thornei* and *P. neglectus*) [15]. Therefore, exploiting genetic variation under abiotic stresses such as drought is needed to further utilize the potential of SHWs.

About 800 quantitative trait loci (QTLs) and marker–trait associations (MTAs) have been reported for drought tolerant traits (agronomic, physiological, root, and yield-related traits) using bi-parental mapping (~691 QTLs) and genome-wide association studies (GWASs; ~109 MTAs) in wheat [3]. However, only 68 QTLs are major QTLs that explain more than 19% of phenotypic variation [3]. This study was conducted to identify novel genomic regions associated with grain yield (GY) and yield-related traits using GWAS performed using 35,648 genotyping-by-sequencing (GBS)-derived single nucleotide polymorphisms (SNPs) in 123 SHWs grown under two drought-stressed growing seasons (2016 and 2017) in Konya, Turkey. Subsequently, the underlying genes for the MTAs identified were investigated for their potential role in drought stress using the functional annotations. To the best of our knowledge, this is the first report on GWAS on GY and yield-related traits under drought stress in SHWs. The results from this study will be a valuable resource for the genetic improvement of GY and yield-related traits in drought stress, introgression of desirable genes from SHWs into elite wheat germplasm, genomic selection, and marker-assisted selection in the breeding program.

## 2. Results and Discussion

### 2.1. Weather Conditions

The mean monthly air temperatures were similar at Konya in both growing seasons with 13 °C in 2015–2016 and 12 °C in 2016–2017 compared to the 25-year mean monthly air temperature (11 °C) in Turkey (Table 1). The total rainfall during 2016–2017 (243 mm) was slightly higher than that during 2015–2016 (222.4 mm) growing season. Total rainfalls during the wheat-growing season (September–July) were 48.9% lower in 2015–2016 and 44.2% lower in 2016–2017 compared to the 25-year mean total rainfall (435.1 mm) in Turkey. Although winter wheat water requirements are higher from mid-March to mid-June (from the spring tillering period to the mid-grain filling period), rainfalls were lower in both years compared to the 25-year mean rainfall. The plants were exposed to drought stress from tillering through grain filling. Hence, the results from the present study can be used to understand the effects and genetics of drought in SHW.

### 2.2. Phenotypic Variation for Yield and Yield-Related Traits

A combined analysis of variance (ANOVA) across years identified significant cross-over genotype × year interaction for all traits except for flag leaf width (FLW) and stem diameter (STMDIA) (Table S4). Therefore, analysis of variance was computed for both years separately and the results indicated that the SHWs showed significant variation for GY and yield-related traits in each year (Table 2). For instance, GY ranged from 200 g⋅m^−2^ to 341 g⋅m^−2^ with an average yield of 259 g⋅m^−2^ in 2016 and from 241 g⋅m^−2^ to 392 g⋅m^−2^ with an average yield of 290 g⋅m^−2^ in 2017 (Table 2). The large variation among the traits in each year can be attributed to the collection of diverse accessions of SHWs from different countries and different genetic backgrounds [11,14].

Broad-sense heritability (*H^2^*) ranged from low to high (Table 2). Low to moderate *H^2^*was observed for GY (0.32–0.56), biomass weight (BMWT; 0.39–0.63), FLW (0.49–0.67), and root length (RTLN; 0.31–0.60); moderate *H^2^*was observed for harvest index (HI; 0.63–0.64) and STMDIA (0.57–0.63); moderate to high *H^2^*was observed for flag leaf length (FLLN; 0.53–0.91), flag leaf area (FLA; 0.52–0.85) and awn length (AWNLN; 0.61–0.95); and high *H^2^* was observed for thousand kernel weight (TKW; 0.75–0.90) and grain volume weight (GVWT; 0.76–0.91), indicating the genetic instability of these traits across years under drought stress. Similar *H^2^* for most of these traits have been observed in previous studies [17,18,19,20,21,22,23,24,25,26].

### 2.3. Principal Component Analysis and Phenotypic Correlation

**Figure 1:**
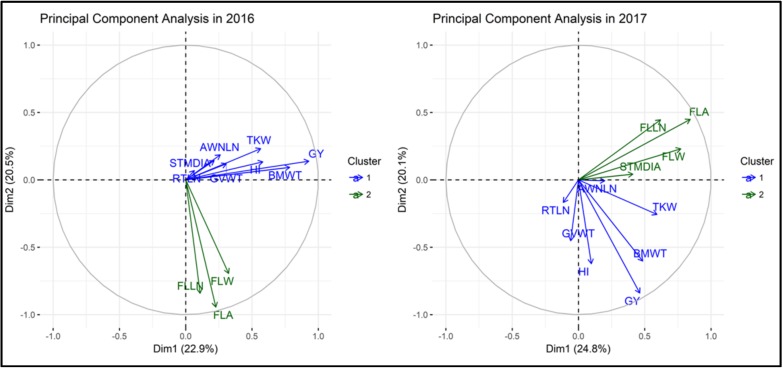
Principal component bi-plot analysis of 123 drought-stressed synthetic hexaploid wheat grown in two seasons (2016 and 2017) in Konya, Turkey. AWLN, awn length; BMWT, biomass weight; FLA, flag leaf area; FLLN, flag leaf length; FLW, flag leaf width; GVWT, grain volume weight; GY, grain yield; HI, harvest index; RTLN, root length; STMDIA, stem diameter; and TKW, thousand kernel weight.

Principal component (PC) bi-plot analysis showed the association between GY and yield-related traits based on correlation matrix (Figure 1). The first two PCs that explained 43.4% (2016) and 44.9% (2017) of variation better explained the relationship between traits in the two-dimensional space. In the PC biplot, we observed two distinct groupings. The first one comprised of GY, HI, BMWT, TKW, GVWT, AWNLN, and RTLN whereas the second one had FLLN, FLW, and FLA (Figure 1). The traits grouping with GY are the more important traits for improving GY in drought-stressed conditions. The association observed in the PC biplot was supported by the significant correlations of GY with BMWT, HI, TKW, and GVW in both years (Figure S1). Similar correlations for these traits were observed in the previous studies [18,27,28,29,30,31,32].

### 2.4. Population Structure and Genome-Wide Association Study

Population structure analysis of 123 SHWs was performed using 35,648 SNPs [filtered for minor allele frequency (MAF) > 0.05 and missing data < 20%] using the Bayesian clustering algorithm implemented in Structure software and the results showed that these lines were divided into three subgroups (Figure S2 and Table S1). The details of the population structure and genetic diversity of these SHWs have been previously reported in Bhatta et al. [11].

**Figure 2:**
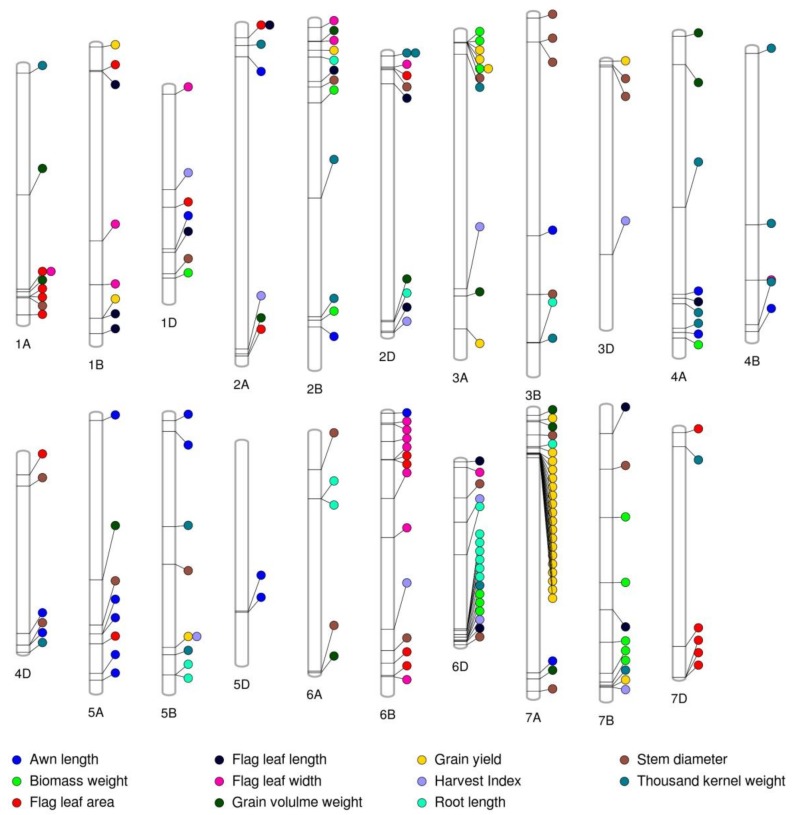
Significant markers trait associations identified on each chromosome for grain yield and yield-related traits obtained from the genome-wide association study of 123 synthetic hexaploid wheats grown in 2016 and 2017 in Konya, Turkey.

The GWAS identified novel genomic regions for GY and yield-related traits and the MTA explained the high phenotypic variance. The Fixed and random model Circulating Probability Unification algorithm (FarmCPU), with kinship, population structure (Q) or PC, best linear unbiased predictors (BLUPs) for each trait, and 35,648 GBS-derived SNPs was used to identify MTAs. The GBS-derived SNPs were well distributed across each of the chromosome (Figure S3). We identified 194 MTAs distributed across 21 chromosomes for GY and yield-related traits with phenotypic variance explained (PVE) ranging from 1.1% to 32.3% (Figure 2 and Figure S4, Table S5). The highest number of MTAs was observed for GY (29), followed by STMDIA (23), FLA (20), and TKW (20) while the lowest MTAs were observed for HI (10) (Figure 2). Of the 194 MTAs, 75 MTAs were detected on the A genome, with 66 MTAs on the B genome, and 53 MTAs on the D-genome. The highest MTAs were present on chromosome 7A (26 MTAs) and the lowest MTAs were present on chromosome 3D (four MTAs) (Figure 2). Most of the MTAs identified in the present study were year-specific, suggesting the influence of genotype × environment interaction on the phenotype of the traits measured in two years. However, 120 of the 194 significant SNPs were in 83 genes and 45 of these MTAs were present within genes and their annotations suggested their potential role in drought stress. This result further provided confidence that the MTAs identified in the study are likely reliable MTAs (Table S5).

#### 2.4.1. Grain Yield

The 29 MTAs for GY were observed in 29 different genomic regions on seven chromosomes including 1B, 2B, 3A, 3D, 5B, 7A, and 7B with PVE ranging from 7.6% to 17.9% (Figure 2, and Table S5). Earlier studies have reported QTLs/MTAs for GY on wheat chromosomes 1B [5,17,19,33], 2B [17,19,29,33,34], 3A [17,30,33,34], 3D [33], 5B [5,28,30,31,33,35], 7A [20,27,30,33], and 7B [17,30,33]. However, it is difficult to align our findings with previous studies due to the use of different marker systems [90K SNP, short sequence repeat (SSR), diversity arrays technology (DART) marker vs. GBS-derived SNP marker], absence of precise location information in published studies, or the use of a different version of the reference wheat genome in previous studies than the International Wheat Genome Sequencing Consortium (IWGSC) RefSeq v1.0. However, identification of several MTAs on the same chromosome as earlier studies provided increased confidence on these associations.

**Figure 3:**
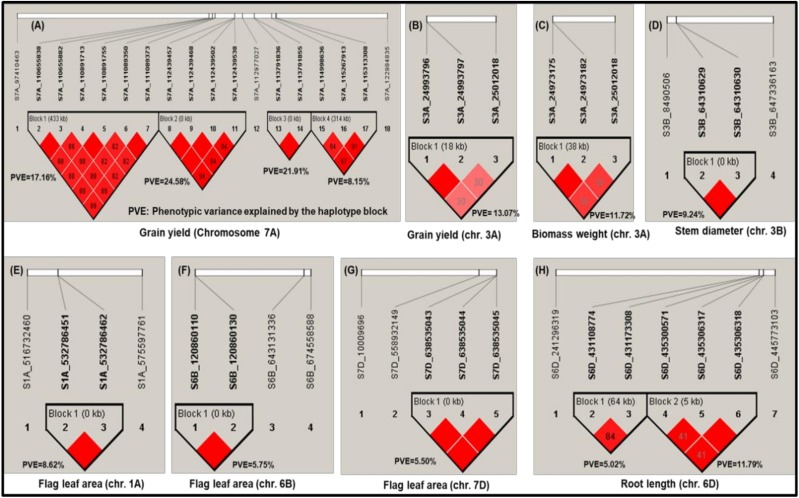
Linkage disequilibrium (LD) values (R^2^) and haplotype blocks with significant marker–trait associations (MTAs; ≥2) observed (**A**) on chromosome 7A for GY, (**B**) on chromosome 3A for GY, (**C**) on chromosome 3A for BMWT, (**D**) on chromosome 3B for STMDIA, (**E**) on chromosome 1A for FLA, (**F**), on chromosome 6B for FLA, (**G**) on chromosome 7D for FLA, and (**H**) on chromosome 6D for RTLN and phenotypic variance explained (PVE) by each haplotype block. Dark red color represents the strong LD whereas light red color represents the weak LD between pairs of MTAs.

The present study identified four major haplotype blocks (from 19 bp to 433 kb) on chromosome 7A with two to six SNPs associated with GY in 2016 (Figure 3). First haplotype block consisted of six MTAs within the 433 kb range, second haplotype block consisted of four MTAs within the 81 bp range, third haplotype block consisted of two MTAs within the 19 bp range, and fourth haplotype block consisted of three MTAs within the 314 kb range. The PVE on GY by the first, second, third, and fourth haplotype blocks were 17.2%, 24.6%, 21.9%, and 8.2%, respectively.

One MTA (S7A_112977027; 112977027 bp) present in-between the second (537 kb away) and third (837 kb) haplotype blocks was within the gene, TraesCS7A01G158200.1, and PVE on GY was 12.8% (Table S5). This gene was annotated as a member of sentrin-specific protease of Ubiquitin-like Protease 1 (*Ulp1*) gene family (Table 3). The *Ulp1* is a small ubiquitin related modifier (SUMO)-specific protease that affects several important biological processes in plants including response to abiotic stress [21]. It has been shown to play a role in drought tolerance in Arabidopsis (*Arabidopsis thaliana*) [36] and rice (*Oryza sativa*) [22,37]. This makes this MTA interesting and a stronger candidate for future functional validation studies.

Another major haplotype block (18 kb) of three MTAs was observed on chromosome 3A in 2017 (Figure 3) and the PVE on GY by this haplotype block was 13.1% (Figure 3). The chromosome 3A is known to be an important chromosome that contains useful QTLs for GY and yield-related traits [17,18,19,20,21,22,30,31,32,33,34,35,36,37,38] and the haplotype block identified will have a significance in the crop improvement program. All three MTAs present in this haplotype block of chromosome 3A were found in the gene, TraesCS3A01G047300 (Table S5), which was annotated as a member of the F-box gene family (Table 4). These three SNPs were indicated as having a moderate impact on the protein as they resulted in a missense mutation and caused an amino acid change. Such changes may alter the function of the protein [39], which makes this F-box gene a strong candidate for future functional characterization studies under drought tolerance in wheat. The F-box proteins are known to regulate many important biological processes, such as embryogenesis, floral development, plant growth and development, biotic and abiotic stresses, hormonal responses, and senescence [39]. Two other MTAs observed on chromosome 3A and 3D were present within genes (F-box family protein: TraesCS3A01G445100 and disease resistance protein RPM1: TraesCS3D01G002700) that have been previously reported to be involved in drought tolerance [39,40] (Table 4).

The GY haplotype blocks and other MTAs identified in the present study have not been mapped to date and four MTAs were in the genes, of which functional annotations suggested that they are likely involved in drought tolerance. This result implied that haplotype blocks observed on chromosome 3A (3 MTAs) and 7A (16 MTAs), and one MTA on chromosome 3D (1) for GY are novel and may potentially be used in a marker-assisted breeding program, focusing on improving drought tolerance in wheat after validating them in different populations and environments.

#### 2.4.2. Harvest Index

A total of 10 SNPs significantly associated with HI were identified on chromosomes 1D, 2A, 2D, 3A, 3D, 5B, 6B, 6D, and 7B (Figure 2) with PVE ranging from 2.2% to 18.7% (Table S5). Previous studies have reported QTLs/MTAs responsible for HI on chromosomes 2D [27], 3A [27,30], 6B [41], and 7B [30]. To the best of our knowledge, the six MTAs identified for HI on chromosomes 1D, 2A, 3D, 5B, and 6D have not been reported and they are potentially novel MTAs responsible for HI.

Six MTAs for HI detected on chromosomes 2A, 3A, 6B, 6D, and 7B were found in genes and two of these genes have annotations suggesting their involvement in drought stress (Table 3 and Table S5). The two genes are WRKY transcription factor (TraesCS3A01G343700) found on chromosome 3A and cytochrome P450 (TraesCS6D01G170900.1) found on chromosome 6D. The role of WRKY transcription factor is well known in abiotic stresses including drought tolerance [42,43]. The cytochrome P450 genes are a large superfamily of enzymes and are involved in many metabolic pathways including drought tolerance in rice [44] and Arabidopsis [45,46]. The multi-trait marker associated with GY and HI was located on chromosome 5B (S5B_598463062) with PVE ranging from 15.9% to 18.7% (Table S5).

#### 2.4.3. Biomass Weight

The 15 MTAs responsible for BMWT were identified on chromosomes 1D, 2B, 3A, 4A, 6D, and 7B (Figure 2) with PVE ranging from 4.9% to 14.4% (Table S5). Previous studies have reported QTLs/MTAs responsible for BMWT on chromosomes 1D [30,41], 2B [27], 6D [27], and 7B [30]. The four MTAs identified for BMWT on chromosome 3A and 4A have not been reported and they are potentially novel MTAs responsible for BMWT.

A novel haplotype block (38 kb) of three SNPs on chromosome 3A associated with BMWT was identified in 2017 (Figure 3) with PVE by the haplotype block of 11.7%. This MTA (S3A_25012018) was also associated with GY and PVE ranged from 12.7% (GY) to 14.4% (BMWT).

All three MTAs present in this haplotype block were within genes (Table S5) and one of the genes had annotations suggesting its involvement in drought tolerance was an F-box family protein (TraesCS3A01G047300) (Table 4) [39]. Excluding MTAs on haplotype block, eight MTAs for BMWT detected on chromosomes 1D, 2B, 6D, and 7B were found in genes (Table S5) and two of the genes had annotations suggesting its involvement in drought stress (Table 3 and Table 4). The genes associated with two MTAs are F-box family protein (TraesCS7B01G242600) [39] and protein DETOXIFICATION containing multi-antimicrobial extrusion protein (MatE) (TraesCS1D01G357500) [23].

#### 2.4.4. Thousand Kernel Weight

A total of 20 MTAs responsible for TKW were detected in 19 different genomic regions on chromosomes 1A, 2A, 2B, 2D, 3A, 3B, 4A, 4B, 4D, 5B, 6D, 7B, and 7D (Figure 2) with PVE ranging from 1.6% to 22.2% (Table S5). Earlier studies have reported QTLs/MTAs for TKW on chromosomes 1A [19,24,29,30], 2A [20], 2B [20,29,30], 2D [19], 3A [24,25,29], 3B [20,26], 4B [5], 5B [24], 7B [30], and 7D [20]. In the present study, only one MTA (S2D_7309581) responsible for TKW was detected on chromosome 2D in both years and assumed to be a stable MTA, because this MTA was detected despite significant genotype x year interaction. The five MTAs identified for TKW on chromosomes 4A, 4D, and 6D have not been previously reported and they are potentially novel MTAs responsible for TKW.

Twelve MTAs responsible for TKW detected on chromosomes 2A, 2B, 3A, 3B, 4A, 4B, 5B, and 6D were found in genes (Table S5) and five of these genes had annotations suggesting their involvement in drought stress (Table 3 and Table 4). The genes associated with MTAs involved in drought tolerance are F-box family protein (chromosome 3A; TraesCS3A01G047300) [39], protein kinase family protein (chromosome 4A; TraesCS4A01G347600 and chromosome 6D; TraesCS6D01G360800) [47], cytochrome P450 family protein (chromosome 4D; TraesCS4D01G364700) [44,45,46], and zinc finger (C3HC4-type RING finger) family protein (chromosome 4B; TraesCS4B01G344200.1) [48,49,50]. The SNP S4D_509427923 was annotated as a missense variant and thus may have a moderate impact on the protein function (Table S5).

#### 2.4.5. Grain Volume Weight

Thirteen MTAs responsible for GVWT were identified on chromosomes 1A, 2A, 2B, 2D, 3A, 4A, 5A, 6A, and 7A (Figure 2) with PVE ranging from 1.3% to 16.2% (Table S5). Earlier studies have reported QTLs/MTAs for GVWT on chromosomes 1A [33], 2A [33,51], 2B [33,51], 2D [33,51], 5A [33] and 7A [33,51]. Four MTAs identified for GVWT on chromosomes 3A, 4A, and 6A have not been previously reported and they are potentially novel MTAs responsible for GVWT.

Eight MTAs responsible for GVWT detected on chromosomes 1A, 2A, 2B, 4A, 6A, and 7A were found in genes (Table S5) and two of these genes had annotations suggesting their involvement in drought stress (Table 4). The genes associated with two MTAs involved in drought tolerance are cytochrome P450 (TraesCS1A01G334800) [44,45,46] on chromosome 1A and microtubule-associated protein family protein (TraesCS4A01G074200.2) [52] on chromosome 4A.

#### 2.4.6. Awn Length

Twenty MTAs responsible for AWNLN were observed on chromosomes 1D, 2A, 2B, 3B, 4A, 4B, 4D, 5A, 5B, 5D, 6B, and 7A (Figure 2) with PVE ranging from 1.1% to 20.1% (Table S5). Earlier studies have reported QTLs/MTAs for AWNLN on chromosomes 2A [53,54], 4A [54], 4B [54], 5A [54], and 6B [53,54]. The nine MTAs identified for AWNLN on chromosomes 2B, 3B, 4D, 5B, 5D, and 7A have not been previously reported and they are potentially novel MTAs responsible for AWNLN.

Eleven MTAs responsible for AWNLN detected on chromosomes 1D, 2A, 4D, 5A, 5B, 6B, and 7A were found in genes (Table S5) and four of these genes had annotations suggesting their involvement in drought stress (Table 3 and Table 4). The genes associated with four MTAs involved in drought tolerance are 60S ribosomal protein L18a (chromosome 4D; TraesCS4D01G290700) [55], guanine nucleotide exchange family protein (chromosome 5A; TraesCS5A01G361300) [56], and F-box family protein (chromosome 5B; TraesCS5B01G038700 and chromosome 6B; TraesCS6B01G001000) [39]. It has been reported that the putative 60S ribosomal protein L18a is an upregulated transcript in response to drought stress in ears and silks during the flowering stage in maize [55].

#### 2.4.7. Flag Leaf Length

Thirteen MTAs responsible for FLLN were detected on chromosomes 1B, 1D, 2A, 2B, 2D, 4A, 6D, and 7B (Figure 2) with PVE ranging from 1.58% to 32.3% (Table S5). Previous studies have reported QTLs for FLLN on chromosomes 1B [57,58], 2B [57,58,59,60], 2D [57,61], 4A [57,58,59], and 7B [61]. The four MTAs identified for FLLN on chromosomes 1D, 2A, and 6D have not been previously reported and they are potentially novel MTAs responsible for FLLN.

Eleven MTAs responsible for FLLN detected on chromosomes 1B, 1D, 2B, 2D, 4A, 6D, and 7B were found in genes (Table S5) and four of these genes had annotations suggesting their involvement in drought stress (Table 3 and Table 4). The genes associated with four MTAs involved in drought stress are F-box family protein (chromosome 4A: TraesCS4A01G325200) [39], cytochrome P450 (chromosome 2B; TraesCS2B01G167500 and chromosome 6D; TraesCS6D01G386300) [44,45,46], and Rp1-like protein (chromosome 1B; TraesCS1B01G400600) [40].

#### 2.4.8. Flag Leaf Width

Sixteen MTAs responsible for FLW were detected on chromosomes 1A, 1B, 1D, 2B, 2D, 4B, 6B, and 6D (Figure 2) with PVE ranging from 1.6% to 15.2% (Table S5). Previous studies have found QTLs for FLW on chromosomes 1B [57,60,61], 1D [57,59], 2B [57,59], 2D [57,59,61], 4B [59,60], and 6B [57,58,59]. The two MTAs identified for FLW on chromosomes 1A and 6D have not been previously reported and they are potentially novel MTAs responsible for FLW.

Thirteen MTAs responsible for FLW detected on chromosomes 1A, 1B, 1D, 2D, 4B, 6B, and 6D were found in genes (Table S5) and three of these genes had annotations suggesting their involvement in drought stress (Table 3 and Table 4). The genes associated with three MTAs involved in drought stress are citrate-binding protein (chromosome 1A; TraesCS1A01G326700) [62], F-box family protein (chromosome 6B; TraesCS6B01G042800) [39], and mitochondrial transcription termination factor-like (chromosome 6D; TraesCS6D01G040100) [63]. The SNPs S1A_516732460 and S6D_16376439 were annotated as a missense variant and thus may impact the function of the proteins that are annotated as citrate-binding protein and mitochondrial transcription termination factor-like protein, respectively (Table S5).

#### 2.4.9. Flag Leaf Area

Twenty MTAs responsible for FLW were detected on chromosomes 1A, 1B, 1D, 2A, 2D, 4D, 5A, 6B, and 7D (Figure S2) with PVE ranging from 8.1% to 23.1% (Table S5). Previous studies have reported QTLs for FLA on chromosomes 1B [58,59], 1D [57,59,61], 2A [57,59,61], 2D [57,59,61], 4D [58], 5A [57,58,60,61], 6B [58], and 7D [61]. The four MTAs identified for FLA on chromosome 1A have not been previously reported and they are potentially novel MTAs responsible for FLA.

Three novel haplotype blocks were observed for FLA on chromosomes 1A (two MTAs), 6B (two MTAs) and 7D (three MTAs) with PVE by these haplotype block ranging from 5.5% to 8.6% (Figure 3). Fourteen MTAs responsible for FLA detected on chromosomes 1A, 1B, 1D, 2A, 4D, 5A, 6B, and 7D were found in genes (Table S5) and five of these genes had annotations suggesting their involvement in drought stress (Table 3 and Table 4). The genes associated with five MTAs involved in drought stress are citrate-binding protein (TraesCS1A01G326700), P-loop containing nucleoside triphosphate hydrolases superfamily protein (TraesCS1D01G197200) [64], cytochrome P450 (TraesCS6B01G125900) [44,45,46], and NBS-LRR resistance-like protein [40].

The multi-trait marker associated with FLW and FLA was located on chromosome 1A (S1A_516732460) with PVE ranging from 8.0% to 9.5% (Table S4). Another multi-trait marker associated with FLLN and FLA was located on chromosome 2A (S2A_29874199) with PVE ranging from 23.1% to 32.3% (Table S4). The multi-trait MTA indicates that the related candidate gene may affect multiple traits.

#### 2.4.10. Stem Diameter

In the present study, 23 MTAs responsible for STMDIA were identified on chromosomes 1A, 1D, 2B, 2D, 3A, 3B, 3D, 4D, 5A, 5B, 6A, 6B, 6D, 7A, and 7B (Figure 2) with PVE ranging from 2.7% to 28.8% (Table S5). Earlier study has identified one minor QTL (QSd-3B) for STMDIA on chromosome 3B that explained 8.7% of the phenotypic variance [65]. It means that, 19 MTAs detected on chromosomes 1A, 1D, 2B, 2D, 3A, 3D, 4D, 5A, 5B, 6A, 6B, 6D, 7A, and 7B except chromosome 3B in the present study may potentially be a novel MTAs controlling STMDIA under drought stress.

Four MTAs were detected on chromosome 3B and two of them (1 bp apart) were observed in one haplotype block in 2016 with PVE was 9.2% (Figure 3). Fifteen MTAs for STMDIA detected on chromosomes 1D, 2B, 3A, 3B, 6B, 6D, 7A, and 7B were found in genes (Table S5) and four of these genes had annotations suggesting their involvement in drought stress (Table 3 and Table 4). The genes associated with four MTAs involved in drought stress are leucine-rich repeat receptor-like protein kinase family protein (TraesCS3D01G028500) [66], protein kinase (TraesCS6A01G122200.1) [47], disease resistance protein (NBS-LRR class) family (TraesCS1D01G341500 and TraesCS6B01G346900) [40], and F-box protein family (TraesCS6B01G347000) [39].

#### 2.4.11. Root Length

RTLN is one of the most important traits under drought stress. We have measured RTLN 3–4 days after anthesis (Zadoks 60 growth stage) under the drought-stressed field condition using WinRhizo^®^ (WinRhizo reg. 2009c, Regent Instruments Inc., Quebec City, QC, Canada). This trait is very unique compared to previous studies where they focused on the roots of seedlings [67,68,69,70] rather than direct field-based measurements (labor intensive, time consuming, and expensive). Identification of QTL governing RTLN is very important in wheat, especially for the wheat grown under drought stress. Limited information is available on QTL related to root traits in wheat [67,69,70,71,72].

In the present study, 15 MTAs responsible for RTLN were identified on chromosomes 2B, 2D, 3B, 5B, 6A, 6D, and 7A (Figure 2) with PVE ranging from 5.3% to 18.5% (Table S5). Earlier studies have reported QTLs for RTLN on chromosomes 2B [68], 3B [69], 5B [67,69], 6A [68,69], and 6D [67,70] in hexaploid wheat and on chromosomes 2B [71,72], 3B [72], 6A [72], and 7A [72] in tetraploid wheat. The MTA identified for RTLN on chromosome 2D has not been previously reported and it is potentially novel MTAs responsible for RTLN under drought stress. Furthermore, previous studies identified very few QTLs for RTLN on the D-genome of wheat [67,70]. Therefore, the MTAs (eight MTAs) for RTLN detected on the D-genome of SHWs in the present study are potentially novel.

Seven out of eight MTAs responsible for RTLN were present on chromosome 6D. Two haplotype blocks (the haplotype block1 with a size of 64 kb and the haplotype block2 with a size of 5kb) were identified from five out of seven MTAs for RTLN on chromosome 6D with PVE ranging from 5.0% to 11.8% (Figure 3). One SNP (S6D_435300571) present in the haplotype block2 was found in the gene (TraesCS6D01G332800) with PVE of 13.0%. The gene associated with this SNP is protein detoxification gene-containing multi-antimicrobial extrusion protein (MatE) (Table 3) and has been reported to be expressed mainly in the root than shoots under drought stress [73]. For instance, *MatE* family genes such as *HvAACT1* in barley [74] and *TaMate* in wheat [75], encode proteins that are primarily localized to root epidermis cells [74] and required for external resistance [23]. In the present study, this gene was also significantly associated with BMWT on chromosome 1D. This result implied that that this gene plays an important role for RTLN and BMWT in drought-stressed conditions.

Excluding MTAs on haplotype block, eight MTAs for RTLN detected on chromosomes 2D, 3B, 5B, 6A, and 7A were found in genes (Table S5) and four of these genes had annotations suggesting their involvement in drought stress (Table 3 and Table 4). The genes associated with four MTAs involved in drought stress are GRAM domain-containing protein/ABA-responsive (TraesCS5B01G502200) [76,77,78,79,80,81], phosphatase 2C family protein (TraesCS7A01G143200.2) [82], and disease resistance protein RPM1 (TraesCS2D01G541000.1) [40]. The SNPs S7A_94404310 was annotated as a missense variant and thus may have a moderate impact on the protein function (Table S5).

### 2.5. Potential Candidate Gene Annotations Affecting Yield and Yield-related Traits under Drought Stress

**Figure 4:**
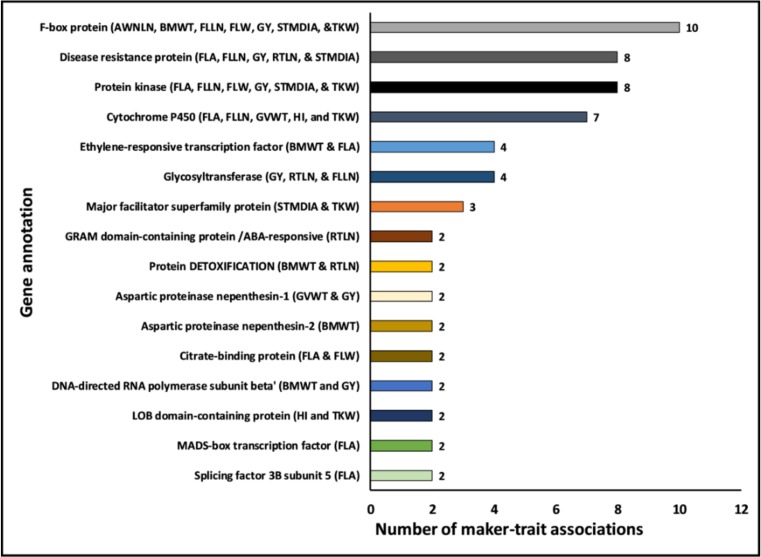
Potential candidate gene functions harboring SNPs affecting yield and yield-related traits under drought stress. The count of marker–trait associations (for either single or multiple traits) located within genes that have the same gene annotation is shown. AWLN, awn length; BMWT, biomass weight; FLA, flag leaf area; FLLN, flag leaf length; FLW, flag leaf width; GVWT, grain volume weight; GY, grain yield; HI, harvest index; RTLN, root length; STMDIA, stem diameter; and TKW, thousand kernel weight.

This study identified ~194 MTAs present on different chromosomes and associated with multiple traits. These 62 MTAs were associated with either the same trait in multiple years (MTA stability in different environments) or multiple traits within the same year or across years (suggesting epistasis) (Table S5). Additionally, ~45 of the MTAs were present in genes with annotations relevant to the respective trait under drought stress (Table 3 and Table 4). Interestingly, we noticed MTAs associated with the same or related traits were located within genes that had the exact same annotation (Figure 4; and Table S6). For instance, some of the MTAs for GY (2 MTAs), BMWT (2), TKW (1), AWNLN (2), FLLN (1), FLW (1), and STMDIA (1) were located within genes annotated as F-box family protein (Table S6). Similarly, the genes annotated as cytochrome P450 harbored MTAs for HI (1), TKW (1), FLA (2), GVWT (1), and FLLN (2). Additional examples are provided in Figure 4 and Table S6 in Supplementary Materials. This result indicated the likely gene families that are important for GY and yield-related traits under drought stress.

## 3. Materials and Methods

### 3.1. Site Description

A field experiment was conducted during two growing seasons (2016 and 2017) under drought stressed conditions (rainfed) at the research farm located at the Bahri Dagdas International Agricultural Research Institute in Konya, Turkey (37°51′15.894″ N, 32°34′3.936″ E; Elevation = 1021 m). This site was characterized by a low precipitation (below 300 mm), low humidity, and slightly alkaline clay loam soil [83].

### 3.2. Plant Materials and Experimental Design

One hundred twenty-three SHWs developed from two introgression programs were used (Table S1). The first group was developed by Kyoto University, Japan from one spring durum (‘Langdon’) parent crossed with 14 different *Ae. tauschii* accessions resulting in 14 different lines (Table S2). The remaining 109 lines were the second group of synthetics that were developed by International Maize and Wheat Improvement Center (CIMMYT) from the six durum parents crossed with 11 different *Ae. tauschii* accessions mainly from the Caspian Sea Basin area. Initially, 13 crosses among six winter durum wheats were involved in the creation of 13 different winter-type synthetics. However, due to the partial sterility, segregation, and continuous selection in the early generation, 109 lines were selected as unique lines because of their differences in phenotype [14] and their kinship values [11]. The synthetic genotypes used in the present study are unique and have been developed recently and tested for their agronomic traits [14], genetic diversity, and population structure [11]. The detailed information of these SHWs were provided in Bhatta et al. [11].

The experimental design was an augmented design (plot size: 1.2 m × 5 m) with replicated checks (‘Gerek’ and ‘Karahan’) in the 2016 growing season and modified alpha-lattice design (plot size: 1.2 m × 5 m) including 123 SHWs and replicated checks (‘Gerek’ and ‘Karahan’) with two replications in the 2017 growing season. The SHWs were planted on 20 September in 2015 and harvested on 18 July 2016 for the 2016 growing season, whereas the SHWs were planted on 15 September in 2016 and harvested on 21 July 2017 for the 2017 growing season.

### 3.3. Trait Measurements

The GY was obtained by harvesting four middle rows of 1.008 m^2^ (i.e., 84 m × 120 m) and reported in g⋅m^−2^. The HI, BMWT, TKW, GVWT, AWNLN, FLLN, FLW, and FLA (0.8 × FLLN × FLW) were measured using previously reported protocols [18,32,59,61]. The STMDIA was measured from five randomly selected plants per plot using a digital Vernier caliper at the second internode from the soil surface at physiological maturity. The RTLN was measured from randomly selected three plants per plot after 3–4 days of flowering (Zadoks 60 growth stage) using WinRhizo software (WinRhizo reg. 2009c, Regent Instruments Inc., Quebec City, QC, Canada).

### 3.4. Phenotypic Data Analysis

A combined ANOVA was performed using the following model:

(1) $$\;y_{\mathrm{ijklmn}}=\mu +Yr_{i}+{R(Yr)}_{\mathrm{ji}}+{B(R(Yr))}_{\mathrm{kji}}+C_{l}+G_{m(\mathrm{kji})}+{\mathrm{GXY}r}_{\mathrm{mi}}+e_{\mathrm{ijklmn}}\;$$

where *y*_ijklm_ is the GY and yield-related trait; *μ* is overall mean; *Y*r_i_ is the effect of ith year; *R*(*Y*r)_ji_ is the effect of jth replication within the ith year; *B*(*R*(*Y*r))_kji_ is the effect of kth incomplete block within jth replication of ith environment; *C*_l_ is the lth checks; *G*_mkji_ is the effect of mth genotypes (new variable, where check is coded as 0 and entry is coded is 1 and the genotype is taken as a new variable × entry) within the kth incomplete block of jth replication in the ith year; *GXY*r_mi_ is the interaction effect of mth genotype and ith year; and e_ijklmn_ is the residual. In the combined ANOVA, year and check were assumed as fixed effects, whereas genotype, genotype × year interaction, replication nested within a year, and incomplete block nested within replications were assumed as random effects.

Individual analyses of variance were performed because most of the traits had highly significant genotype × year interaction and therefore will be discussed hereafter. An augmented design was analyzed using the following model for the estimation of BLUPs in the year 2016:

(2) $$\;y_{\mathrm{ijkl}}=\mu +B_{i}+C_{j}+G_{k(i)}+e_{\mathrm{ijkl}}\;$$

where *y*_ijkl_ is the trait, *μ* is the overall mean; *B*_i_ is the effect of ith incomplete block; *C*_j_ is the jth check; *G*_k(i)_ (new variable, where check is coded as 0 and entry is coded as 1 and genotype is taken as a new variable × entry) is the effect of kth genotypes within the ith block; e_ijkl_ is the residual. In ANOVA calculated for the 2016 datasets, the check was assumed as a fixed effect, whereas genotype and incomplete block were assumed as random effects.

An alpha (α) lattice design with two replications was analyzed using the following model for the estimation of BLUPs in the year 2017:

(3) $$\;y_{\mathrm{ijkl}}=\mu +R_{i}+{B(R)}_{\mathrm{ji}}+C_{k}+G_{l(\mathrm{ji})}+e_{\mathrm{ijkl}}\;$$

where *y*_ijk_ is the trait, *μ* is the overall mean; *R*_i_ is the effect of ith replication; *B*(*R*)_ji_ is the effect of jth block within the ith replication; *C*_k_ is the kth checks; *G*_lji_ (new variable, where check is coded as 0 and entry is coded as 1 and the genotype is taken as a new variable × entry) is the effect of kth genotypes within jth incomplete block of ith replication; e_ijkl_ is the residual. In ANOVA calculated for the 2017 datasets, the check was assumed as a fixed effect, whereas genotype, replication, and incomplete block nested within replication were assumed as random effects.

All phenotypic data were analyzed using PROC MIXED in SAS 9.4 (SAS Institute Inc., Cary, NC) [84] using the restricted maximum likelihood (REML) approach unless mentioned otherwise.

Broad-sense heritability for each trait in each year was calculated based on entry mean basis using Equations (4–6) for 2016, 2017, and combine0d experiments, respectively:

(4) $$\;H^{2}=\frac{\sigma^{2}g}{\sigma^{2}g+\sigma^{2}e}\;$$

(5) $$\;H^{2}=\frac{\sigma^{2}g}{\sigma^{2}g+\frac{\sigma^{2}e}{r}}\;$$

(6) $$\;H^{2}=\frac{\sigma^{2}g}{\sigma^{2}g+\frac{\sigma^{2}yr}{n}+\frac{\sigma^{2}gxyr}{nr}}\;$$

where σ^2^_g_, σ^2^_yr_, σ^2^_gxyr_, and σ^2^_e_ are the variance components for genotype, year, genotype × year, and error, respectively, and n and r are the numbers of years and replications, respectively.

Pearson’s correlation of GY and yield-related traits was calculated based on BLUPs for each trait in each year using PROC CORR in SAS. The PC biplot analysis (PCA-biplot) was performed based on the correlation matrix to avoid any variation due to the different scales of the measured variables using ‘factoextra’ package in R software [85].

### 3.5. Genotyping and SNP Discovery

Genomic DNA was extracted from two to three fresh young leaves of 14-day-old seedlings using BioSprint 96 Plant Kits (Qiagen, Hombrechtikon, Switzerland), as described in Bhatta et al. [11]. The GBS libraries were constructed in 96-plex following digestion with two restriction enzymes, PstI and MspI [86] and pooled libraries were sequenced using Illumina, Inc. (San Diego, CA, USA) next-generation sequencing platforms at the Wheat Genetics Resource Center at Kansas State University (Manhattan, KS, USA). SNP calling was performed using TASSEL v. 5.2.40 GBS v2 Pipeline (available online: https://bitbucket.org/tasseladmin/tassel-5-source/wiki/Tassel5GBSv2Pipeline) [87] with physical alignment to the Chinese Spring genome sequence (RefSeq v1.0) made available by the IWGSC [88] using default settings with the one exception that the number of times for a GBS tag to be present and included for SNP calling was changed from the default value of 1 to 5 to increase the stringency in SNP calling. The identified SNPs with MAF less than 5% and missing data more than 20% were removed from the analysis. All lines had missing data less than 20% and none of them were dropped due to missing percentage-filtering criterion. The GBS-derived SNPs are provided in Table S3 in Supplementary Materials.

### 3.6. Population Structure and Genome-Wide Association Study Analysis

Population structure of 123 genotypes was assessed using the Bayesian clustering algorithm in the program STRUCTURE v 2.3.4 (available online: https://web.stanford.edu/group/pritchardlab/structure_software/release_versions/v2.3.4/html/structure.html) [89] and principal component (PC) analysis using TASSEL (available online: http://www.maizegenetics.net/tassel) [90], as described in Bhatta et al. [11].

Many GWASs were previously performed using the mixed linear model (MLM), where the population structure (Q) or PC was set as a fixed effect and kinship (K) as a random effect to control false positives [91,92]. However, the MLM may lead to confounding between population structure, kinship, and quantitative trait nucleotides (QTNs) that results in false negatives due to model overfitting [78]. Recently, the multilocus mixed model (MLMM), which tests multiple markers simultaneously by fitting pseudo QTNs, in addition to testing markers in stepwise MLM, has been proposed, which is advantageous over conventional GLM and MLM testing one marker at a time [78]. One of the examples of recently popular GWAS analysis algorithm that is based on MLMM is FarmCPU [78,79]. The FarmCPU uses a fixed effect model (FEM) and a random effect model (REM) iteratively to remove the confounding between testing markers and kinship that results in false negatives, prevents model overfitting, and control false positives simultaneously [78]. Therefore, GWAS was performed on the adjusted BLUPs for each trait in each year to identify SNPs associated with GY and yield-related traits in SHWs using FarmCPU with population structures (Q_1_ and Q_2_) or first three principal components (PC_1_, PC_2_, and PC_3_) as covariates by looking at the model fit using Quantile-Quantile (Q-Q) plots and FarmCPU-calculated kinship [78] implemented in MVP R software package (available online: https://github.com/XiaoleiLiuBio/MVP). A uniform suggestive genome-wide significance threshold level of *p*-value = 9.99 × 10^-5^ (−log_10_*p* = 4.00) was selected for MTAs considering the deviation of the observed test statistics values from the expected test statistics values in the Q-Q plots [28,80] from the two-year results of the present study.

### 3.7. Haplotype Block Analysis

Haplotype blocks with linkage disequilibrium (LD) values (squared correlation coefficient between locus allele frequency; R^2^ > 0.2) in adjacent regions (<500 kb) of significant MTAs were visualized and plotted using default parameters (Hardy–Weinberg *p*-value cut off at 1% and MAF > 0.001) of Haploview software (available online: https://www.broadinstitute.org/haploview/haploview) [81]. PVE by each haplotype block on the trait of interest was calculated using multiple regression analysis that accounted for the population structure by removing the haplotype allele of less than 5% in SAS using PROC REG.

### 3.8. Putative Candidate Gene Analysis

The genes underlying the MTAs and subsequently their annotations were retrieved using a Perl script and the IWGSC RefSeq v1.0 annotations [88] provided for the Chinese Spring. The underlying genes were further examined for their association with GY and yield-related traits under drought stress using previously published literature. Additionally, the SnpEff program (available online: http://snpeff.sourceforge.net/) was used for SNP annotation and predicting the effects of SNPs on the protein function. The MTAs present within genes or flanked (5 kb) by genes were investigated [93].

## 4. Conclusions

The present study showed SHWs have large amounts of genetic variation for GY and yield-related traits. The GWAS in 123 SHWs using 35,648 SNPs identified several novel (90 MTAs: 45 MTAs on the A genome, 11 on the B genome, and 34 on the D-genome) genomic regions or haplotype blocks associated with GY and yield-related traits in drought-stressed conditions. Most of the MTAs (120 MTAs) were present in genes ad several of them (45 MTAs) were annotated with functions related to drought stress. This provided further evidence for the reliability of the MTAs identified. We also identified MTAs on different chromosomes associated with multiple traits but within genes having the same annotation. This resulted in the identification of candidate genes belonging to the same gene family that likely have a major role in affecting GY and yield-related traits under drought stress in SHWs. The large number of MTAs, especially on the D-genome (53 MTAs with 34 MTAs being novel) identified in the present study, demonstrate the potential of SHWs for elucidating the genetic architecture of complex traits and provide an opportunity for further improvement of wheat under rapidly growing drought-stressed environment worldwide.

## Figures and Tables

**Table 1:** Mean monthly temperatures and total monthly rainfalls in two growing seasons (2016 and 2017) and 25-year averaged data in Konya, Turkey.

Month	Konya, 2015–2016	Konya, 2016–2017	Turkey, 1991–2015	Konya, 2015–2016	Konya, 2016–2017	Turkey, 1991–2015
	Temperature (temp) (^°^C) ^a^	Temp (^°^C)	Temp (^°^C) ^b^	Rainfall (mm) ^c^	Rainfall (mm)	Rainfall (mm) ^d^
September	22.8	17.1	19.0	35.8	11.2	23.1
October	15.3	13.2	13.7	34.4	0.0	48.3
November	7.5	4.9	7.0	5.8	16.6	58.0
December	−0.1	0.5	2.1	8.0	26.8	73.0
January	1.6	0.2	0.1	37.0	9.0	65.6
February	4.9	3.4	1.3	0.4	69.2	60.0
March	8.5	8.2	5.3	37.8	31.0	61.6
April	15.2	12.7	10.4	9.6	33.2	62.7
May	18.4	16.7	15.2	38.4	41.2	54.6
June	23.7	24.4	19.5	15.0	4.8	34.7
July	26.6	27.7	22.9	0.2	0.0	15.1
Total/average	13.1	11.7	10.6	222.4	243.0	435.1

^a^ Source: Bahri Dagdas International Agricultural Research Institute. ^b^ Source: http://sdwebx.worldbank.org/climateportal/index.cfm?page=country_historical_climate&ThisCCode=TUR. ^c^ Source: Bahri Dagdas International Agricultural Research Institute. ^d^ Source: http://sdwebx.worldbank.org/climateportal/index.cfm?page=country_historical.

**Table 2:** Phenotypic variation for grain yield and yield-related traits with best linear unbiased predictor values, range, percentage of coefficient of variation (CV), and broad sense heritability (*H^2^*) of 123 synthetic hexaploid wheat grown in two seasons (2016 and 2017) in Konya, Turkey.

Trait	2016				2017			
	Mean	Range	CV	*H^2^*	Mean	Range	CV	*H^2^*
Grain yield (g⋅m^−2^)	259	200–341	9.7	0.32	290	241–392	9.9	0.56
Harvest index	0.4	0.24–0.66	10.9	0.63	0.34	0.27–0.41	6.3	0.64
Biomass weight (g⋅m^−2^)	671	537–827	9.1	0.39	865	684–1098	8.9	0.63
Thousand kernel weight (g)	32.1	24–42	10.5	0.75	41	33–50	8	0.90
Grain volume weight (Kg⋅hL^−1^)	65.6	52–77	7.2	0.91	74	68–77	2.3	0.76
Awn length (cm)	6	2.3–8.6	24.3	0.61	5.6	0.5–8.0	28.3	0.95
Flag leaf length (cm)	22.4	21.8–22.8	0.8	0.91	12	9.9–16.4	7.6	0.53
Flag leaf width (cm)	1	0.96–1.13	2.8	0.67	1	0.9–1.3	6.1	0.49
Flag leaf area (cm^2^)	18.9	17.6–19.7	2.2	0.85	10.1	7.7–14	11.6	0.52
Stem diameter (mm)	2.9	2.4–3.5	6.9	0.57	2.9	2.5–4.0	7.4	0.63
Root length (cm)	393	392–395	0.20	0.6	192.2	72–375	20	0.31

**Table 3:** List of significant markers associated with GY and yield-related traits, favorable alleles (underlined), SNP effects, and drought-related putative genes from genome-wide association study of 123 drought stressed synthetic hexaploid wheats grown in 2016 in Konya, Turkey.

Trait	SNP ^a^	−log_10_ (*p*)	Alleles	SNP Effect	PVE (%)	Gene ID	Annotation
GY	S3A_686179591	4.08	A/G	−14.28	10.7	TraesCS3A01G445100	F-box family protein
GY	S7A_112977027	5.24	A/T	−19.17	12.8	TraesCS7A01G158200.1	Sentrin-specific protease
HI	S3A_593313534	13.56	T/C	0.08	16.0	TraesCS3A01G343700	WRKY transcription factor
HI	S6D_157451060	4.01	A/G	−0.03	6.2	TraesCS6D01G170900.1	Cytochrome P450, putative
HI	S6D_462272376	12.01	G/A	0.02	14.5	TraesCS6D01G382600.1	LOB-domain protein-like
BMWT	S1D_441309135	4.82	C/G	−105.34	14.4	TraesCS1D01G357500.1	Protein DETOXIFICATION
BMWT	S7B_450630784	4.06	A/G	−25.88	10.7	TraesCS7B01G242600.1	F-box family protein
TKW	S2A_47781717	4.52	G/A	0.93	4.2	TraesCS2A01G093500	F-box family protein
TKW	S4A_625466381	4.12	T/G	1.21	15.3	TraesCS4A01G347600	Protein kinase family protein
TKW	S4D_509427923	4.91	C/G	−1.72	10.1	TraesCS4D01G364700	Cytochrome P450 family protein
TKW	S6D_452410667	8.16	A/G	−1.54	17.7	TraesCS6D01G360800	Protein kinase family protein
AWNLN	S4D_461573496	5.71	T/C	0.32	9.0	TraesCS4D01G290700.1	60S ribosomal protein L18a
AWNLN	S5A_562540562	11.67	C/T	−1.71	11.3	TraesCS5A01G361300.1	Guanine nucleotide exchange family protein
FLLN	S1B_667135914	4.38	C/T	−0.16	20.8	TraesCS1B01G447400	Disease resistance protein RPM1
FLW	S6D_16376439	4.85	C/T	−0.02	13.3	TraesCS6D01G040100.1	Mitochondrial transcription termination factor-like
FLA	S1D_278097355	4.74	G/C	0.21	11.5	TraesCS1D01G197200.1	P-loop containing nucleoside triphosphate hydrolases superfamily protein
FLA	S6B_120860110	4.01	G/A	0.17	9.3	TraesCS6B01G125800	Cytochrome P450 family protein, expressed
FLA	S6B_120860130	4.01	A/T	−0.17	9.3	TraesCS6B01G125900	Cytochrome P450 family protein, expressed
STMDIA	S1D_431523575	6.58	A/G	−0.06	10.3	TraesCS1D01G341500	Disease resistance protein (NBS-LRR class) family
STMDIA	S3D_10133372	9.83	G/T	−0.11	8.6	TraesCS3D01G028500.1	Leucine-rich repeat receptor-like protein kinase family protein
STMDIA	S6A_94238211	6.9	T/G	0.06	7.5	TraesCS6A01G122200.1	Protein kinase, putative
RTLN	S5B_669373985	4.62	T/C	0.27	6.9	TraesCS5B01G502200	GRAM domain-containing protein/ABA-responsive
RTLN	S5B_669374027	4.62	T/C	0.27	6.9	TraesCS5B01G502200	GRAM domain-containing protein/ABA-responsive
RTLN	S6D_431108774	4.01	A/G	−0.27	5.8	TraesCS6D01G332800.1	Protein DETOXIFICATION
RTLN	S7A_94404310	4.01	G/A	0.52	7.5	TraesCS7A01G143200.2	Phosphatase 2C family protein

PVE: phenotypic variance explained; GY, grain yield; HI, harvest index; BMWT, biomass weight; TKW, thousand kernel weight; GVWT, grain volume weight; AWNLN, awn length; FLLN, flag leaf length; FLW, flag leaf width; FLA, flag leaf area; STMDIA, stem diameter; RTLN, root length. ^a^ S+chromosome_chromosome position in bp.

**Table 4:** List of significant markers associated with GY and yield-related traits, favorable alleles (underlined), SNP effects, and drought-related putative genes obtained from genome-wide association study of 123 drought stressed synthetic hexaploid wheats grown in 2017 in Konya, Turkey.

Trait	SNP ^a^	−log_10_ (*p*)	Alleles	SNP Effect	PVE (%)	Gene-ID	Annotation
GY	S3A_25012018	4.81	A/G	−20.02	12.7	TraesCS3A01G047300	F-box-domain-containing protein
GY	S3D_1203058	4.12	T/G	14.32	12.8	TraesCS3D01G002700	Disease resistance protein RPM1
BMWT	S3A_25012018	6.08	A/G	−59.44	14.4	TraesCS3A01G047300	F-box-domain-containing protein
TKW	S4B_11905230	8.94	C/G	−1.11	3.9	TraesCS4B01G016200.1	LOB domain-containing protein, putative
TKW	S4B_637722874	5.17	T/C	0.86	2.0	TraesCS4B01G344200.1	Zinc finger (C3HC4-type RING finger) family protein
GVWT	S1A_522189599	4.11	A/G	−0.55	2.5	TraesCS1A01G334800	Cytochrome P450
GVWT	S4A_73454791	5.64	C/T	−0.63	5.5	TraesCS4A01G074200.2	Microtubule associated protein family protein, putative, expressed
AWNLN	S5B_43896804	7.31	C/T	−1.13	6.0	TraesCS5B01G038700	F-box family protein
AWNLN	S6B_643657	10.55	C/T	−1.04	5.7	TraesCS6B01G001000	F-box family protein
FLLN	S1B_631203243	5.26	A/G	−0.21	9.8	TraesCS1B01G400600.1	Rp1-like protein
FLLN	S2B_140752747	4.15	G/C	0.29	1.6	TraesCS2B01G167500.1	Cytochrome P450, putative
FLLN	S2D_642055122	4.12	T/C	0.25	5.9	TraesCS2D01G579800	protein kinase family protein
FLLN	S4A_612662321	5.3	C/T	−0.23	5.3	TraesCS4A01G325200	F-box family protein
FLLN	S6D_463762312	5.72	G/A	0.25	6.0	TraesCS6D01G386300	Cytochrome P450, putative
FLW	S1A_516732460	6.99	A/G	−0.03	9.5	TraesCS1A01G326700.1	Citrate-binding protein
FLW	S6B_26200560	7.13	C/A	0.03	12.3	TraesCS6B01G042800	F-box family protein
FLA	S1A_516732460	6.9	A/G	−0.42	8.0	TraesCS1A01G326700.1	Citrate-binding protein
FLA	S2A_764065400	4.18	G/T	−0.19	3.8	TraesCS2A01G563200	NBS-LRR resistance-like protein
STMDIA	S6B_610963076	5.7	T/G	0.06	7.9	TraesCS6B01G346900-TraesCS6B01G347000	NBS-LRR disease resistance protein and F-box protein-like
RTLN	S2D_620326979	4.22	T/C	192.21	9.9	TraesCS2D01G541000.1	Disease resistance protein RPM1

PVE: phenotypic variance explained; GY, grain yield; HI, harvest index; BMWT, biomass weight; TKW, thousand kernel weight; GVWT, grain volume weight; AWNLN, awn length; FLLN, flag leaf length; FLW, flag leaf width; FLA, flag leaf area; STMDIA, stem diameter; RTLN, root length. ^a^ S+chromosome_chromosome position in bp.

## Supplementary Materials

Supplementary materials can be found at http://www.mdpi.com/1422-0067/19/10/3011/s1.

## Abbreviations

ANOVA	analysis of variance
AWNLN	awn length
BLUP	best linear unbiased predictor
BMWT	biomass weight
FarmCPU	fixed and random model circulating probability unification
FLA	flag leaf area
FLLN	flag leaf length
FLW	flag leaf width
GBS	genotyping-by-sequencing
GVWT	grain volume weight
GWAS	genome wide association study
GY	grain yield
HI	harvest index
IWGC	international wheat genome sequencing consortium
MAF	minor allele frequency
QTN	quantitative trait nucleotide
MLM	mixed linear model
MLMM	multilocus mixed model
MTA	marker trait association
QTL	quantitative trait loci
RTLN	root length
SHW	synthetic hexaploid wheat
SNP	single nucleotide polymorphism
STMDIA	stem diameter
TKW	thousand kernel weight

## References

[1] Kang Y., Khan S., Ma X. (2009). Climate change impacts on crop yield, crop water productivity and food security—A review. Prog. Nat. Sci..

[2] Becker S.R., Byrne P.F., Reid S.D., Bauerle W.L., McKay J.K., Haley S.D. (2016). Root traits contributing to drought tolerance of synthetic hexaploid wheat in a greenhouse study. Euphytica.

[3] Gupta P., Balyan H., Gahlaut V., Gupta P.K., Balyan H.S., Gahlaut V. (2017). QTL analysis for drought tolerance in wheat: Present status and future possibilities. Agronomy.

[4] Smith A.B., Matthews J.L. (2015). Quantifying uncertainty and variable sensitivity within the US billion-dollar weather and climate disaster cost estimates. Nat. Hazards.

[5] Pinto R.S., Reynolds M.P., Mathews K.L., McIntyre C.L., Olivares-Villegas J.-J., Chapman S.C. (2010). Heat and drought adaptive QTL in a wheat population designed to minimize confounding agronomic effects. Theor. Appl. Genet..

[6] Gummadov N., Keser M., Akin B., Cakmak M., Mert Z., Taner S., Ozturk I., Topal A., Yazar S., Morgounov A. (2015). Genetic gains in wheat in Turkey: Winter wheat for irrigated conditions. Crop J..

[7] Cavanagh C.R., Chao S., Wang S., Huang B.E., Stephen S., Kiani S., Forrest K., Saintenac C., Brown-Guedira G.L., Akhunova A. (2013). Genome-wide comparative diversity uncovers multiple targets of selection for improvement in hexaploid wheat landraces and cultivars. Proc. Natl. Acad. Sci. USA.

[8] Cox T. (1997). Deepening the wheat gene pool. J. Crop Prod..

[9] Dreisigacker S., Kishii M., Lage J., Warburton M. (2008). Use of synthetic hexaploid wheat to increase diversity for CIMMYT bread wheat improvement. Aust. J. Agric Res..

[10] Ogbonnaya F. C., Abdalla O., Mujeeb-Kazi A., Kazi A.G., Xu S.S., Gosman N., Lagudah E.S., Bonnett D., Sorrells M.E., Tsujimoto H. (2013). Synthetic hexaploids: Harnessing species of the primary gene pool for wheat improvement. Plant Breed. Rev..

[11] Bhatta M., Morgounov A., Belamkar V., Poland J., Baenziger P.S. (2018). Unlocking the novel genetic diversity and population structure of synthetic hexaploid wheat. BMC Genom..

[12] Zegeye H., Rasheed A., Makdis F., Badebo A., Ogbonnaya F.C. (2014). Genome-wide association mapping for seedling and adult plant resistance to stripe rust in synthetic hexaploid wheat. PLoS ONE.

[13] Das M.K., Bai G., Mujeeb-Kazi A., Rajaram S. (2016). Genetic diversity among synthetic hexaploid wheat accessions (*Triticum aestivum*) with resistance to several fungal diseases. Genet. Resour. Crop Evol..

[14] Morgounov A., Abugalieva A., Akan K., Akın B., Baenziger S., Bhatta M., Dababat A.A., Demir L., Dutbayev Y., El Bouhssini M. (2018). High-yielding winter synthetic hexaploid wheats resistant to multiple diseases and pests. Plant Genet. Resour..

[15] Ogbonnaya F.C., Imtiaz M., Bariana H.S., McLean M., Shankar M.M., Hollaway G.J., Trethowan R.M., Lagudah E.S., Van Ginkel M. (2008). Mining synthetic hexaploids for multiple disease resistance to improve bread wheat. Aust. J. Agric Res..

[16] Jighly A., Alagu M., Makdis F., Singh M., Singh S., Emebiri L.C., Ogbonnaya F.C. (2016). Genomic regions conferring resistance to multiple fungal pathogens in synthetic hexaploid wheat. Mol. Breed..

[17] Sehgal D., Autrique E., Singh R., Ellis M., Singh S., Dreisigacker S. (2017). Identification of genomic regions for grain yield and yield stability and their epistatic interactions. Sci. Rep..

[18] Bhatta M., Eskridge K.M., Rose D.J., Santra D.K., Baenziger P.S., Regassa T. (2017). Seeding Rate, genotype, and topdressed nitrogen effects on yield and agronomic characteristics of winter wheat. Crop Sci..

[19] Ogbonnaya F.C., Rasheed A., Okechukwu E.C., Jighly A., Makdis F., Wuletaw T., Hagras A., Uguru M.I., Agbo C.U. (2017). Genome-wide association study for agronomic and physiological traits in spring wheat evaluated in a range of heat prone environments. Theor. Appl. Genet..

[20] Sukumaran S., Lopes M., Dreisigacker S., Reynolds M. (2018). Genetic analysis of multi-environmental spring wheat trials identifies genomic regions for locus-specific trade-offs for grain weight and grain number. Theor. Appl. Genet..

[21] Murtas G. (2003). A Nuclear Protease Required for Flowering-Time Regulation in Arabidopsis Reduces the Abundance of SMALL UBIQUITIN-RELATED MODIFIER Conjugates. Plant Cell.

[22] Choudhary M.K., Basu D., Datta A., Chakraborty N., Chakraborty S. (2009). Dehydration-responsive nuclear proteome of rice (*Oryza sativa* L.) illustrates protein network, novel regulators of cellular adaptation, and evolutionary perspective. Mol. Cell. Proteomics.

[23] Yokosho K., Yamaji N., Fujii-Kashino M., Ma J.F. (2016). Functional Analysis of a MATE gene OsFRDL2 revealed its involvement in Al-induced secretion of citrate, but a lower contribution to Al tolerance in rice. Plant Cell Physiol..

[24] Sun C., Zhang F., Yan X., Zhang X., Dong Z., Cui D., Chen F. (2017). Genome-wide association study for 13 agronomic traits reveals distribution of superior alleles in bread wheat from the Yellow and Huai Valley of China. Plant Biotechnol. J..

[25] Zanke C.D., Ling J., Plieske J., Kollers S., Ebmeyer E., Korzun V., Argillier O., Stiewe G., Hinze M., Neumann K. (2014). Whole genome association mapping of plant height in winter wheat (*Triticum aestivum* L.). PLoS ONE.

[26] Golabadi M., Arzani A., Mirmohammadi Maibody S.A.M., Sayed Tabatabaei B.E., Mohammadi S.A. (2011). Identification of microsatellite markers linked with yield components under drought stress at terminal growth stages in durum wheat. Euphytica.

[27] Kumar N., Kulwal P.L., Balyan H.S., Gupta P.K. (2007). QTL mapping for yield and yield contributing traits in two mapping populations of bread wheat. Mol. Breed..

[28] Sukumaran S., Dreisigacker S., Lopes M., Chavez P., Reynolds M.P. (2015). Genome-wide association study for grain yield and related traits in an elite spring wheat population grown in temperate irrigated environments. Theor. Appl. Genet..

[29] Lozada D.N., Mason R.E., Babar M.A., Carver B.F., Guedira G.B., Merrill K., Arguello M.N., Acuna A., Vieira L., Holder A. (2017). Association mapping reveals loci associated with multiple traits that affect grain yield and adaptation in soft winter wheat. Euphytica.

[30] Neumann K., Kobiljski B., Denčić S., Varshney R.K., Börner A. (2011). Genome-wide association mapping: A case study in bread wheat (*Triticum aestivum* L.). Mol. Breed..

[31] Edae E.A., Byrne P.F., Haley S.D., Lopes M.S., Reynolds M.P. (2014). Genome-wide association mapping of yield and yield components of spring wheat under contrasting moisture regimes. Theor. Appl. Genet..

[32] Bhatta M., Regassa T., Rose D.J., Baenziger P.S., Eskridge K.M., Santra D.K., Poudel R. (2017). Genotype, environment, seeding rate, and top-dressed nitrogen effects on end-use quality of modern Nebraska winter wheat. J. Sci. Food Agric..

[33] Bordes J., Goudemand E., Duchalais L., Chevarin L., Oury F.X., Heumez E., Lapierre A., Perretant M.R., Rolland B., Beghin D. (2014). Genome-wide association mapping of three important traits using bread wheat elite breeding populations. Mol. Breed..

[34] Hoffstetter A., Cabrera A., Sneller C. (2016). Identifying quantitative trait loci for economic traits in an elite soft red winter wheat population. Crop Sci..

[35] Wang S.-X., Zhu Y.-L., Zhang D.-X., Shao H., Liu P., Hu J.-B., Zhang H., Zhang H.-P., Chang C., Lu J. (2017). Genome-wide association study for grain yield and related traits in elite wheat varieties and advanced lines using SNP markers. PLOS ONE.

[36] Catala R., Ouyang J., Abreu I.A., Hu Y., Seo H., Zhang X., Chua N.-H. (2007). The Arabidopsis E3 SUMO Ligase SIZ1 Regulates Plant Growth and Drought Responses. Plant Cell.

[37] Park H.J., Kim W.-Y., Park H.C., Lee S.Y., Bohnert H.J., Yun D.-J. (2011). SUMO and SUMOylation in plants. Mol. Cells.

[38] Ali M.L., Baenziger P.S., Ajlouni Z.A., Campbell B.T., Gill K.S., Eskridge K.M., Mujeeb-Kazi A., Dweikat I. (2011). Mapping QTL for agronomic traits on wheat chromosome 3a and a comparison of recombinant inbred chromosome line populations. Crop Sci..

[39] Lechner E., Achard P., Vansiri A., Potuschak T., Genschik P. (2006). F-box proteins everywhere. Curr. Opin. Plant Biol..

[40] Lee H.-A., Yeom S.-I. (2015). Plant NB-LRR proteins: Tightly regulated sensors in a complex manner. Brief. Funct. Genomics.

[41] Ain Q., Rasheed A., Anwar A., Mahmood T., Imtiaz M., Mahmood T., Xia X., He Z., Quraishi U.M. (2015). Genome-wide association for grain yield under rainfed conditions in historical wheat cultivars from Pakistan. Front. Plant Sci..

[42] He G.-H., Xu J.-Y., Wang Y.-X., Liu J.-M., Li P.-S., Chen M., Ma Y.-Z., Xu Z.-S. (2016). Drought-responsive WRKY transcription factor genes TaWRKY1 and TaWRKY33 from wheat confer drought and/or heat resistance in Arabidopsis. BMC Plant Biol..

[43] Ning P., Liu C., Kang J., Lv J. (2017). Genome-wide analysis of WRKY transcription factors in wheat (*Triticum aestivum* L.) and differential expression under water deficit condition. PeerJ.

[44] Tamiru M., Undan J. R., Takagi H., Abe A., Yoshida K., Undan J. Q., Natsume S., Uemura A., Saitoh H., Matsumura H., Urasaki N., Yokota T., Terauchi R. (2015). A cytochrome P450, OsDSS1, is involved in growth and drought stress responses in rice (*Oryza sativa* L.). Plant Mol. Biol..

[45] Seki M., Narusaka M., Ishida J., Nanjo T., Fujita M., Oono Y., Kamiya A., Nakajima M., Enju A., Sakurai T. (2002). Monitoring the expression profiles of 7000 Arabidopsis genes under drought, cold and high-salinity stresses using a full-length cDNA microarray: Expression profiling under abiotic stresses. Plant J..

[46] Kushiro T., Okamoto M., Nakabayashi K., Yamagishi K., Kitamura S., Asami T., Hirai N., Koshiba T., Kamiya Y., Nambara E. (2004). The Arabidopsis cytochrome P450 CYP707A encodes ABA 8′-hydroxylases: key enzymes in ABA catabolism. EMBO J..

[47] Yan J., Su P., Wei Z., Nevo E., Kong L. (2017). Genome-wide identification, classification, evolutionary analysis and gene expression patterns of the protein kinase gene family in wheat and Aegilops tauschii. Plant Mol. Biol..

[48] Sakamoto H., Maruyama K., Sakuma Y., Meshi T., Iwabuchi M., Shinozaki K., Yamaguchi-Shinozaki K. (2004). Arabidopsis Cys2/His2-Type zinc-finger proteins function as transcription repressors under drought, cold, and high-salinity stress conditions. Plant Physiol..

[49] Ciftci-Yilmaz S., Morsy M.R., Song L., Coutu A., Krizek B.A., Lewis M.W., Warren D., Cushman J., Connolly E.L., Mittler R. (2007). The EAR-motif of the Cys2/His2-type zinc finger protein Zat7 plays a key role in the defense response of Arabidopsis to salinity stress. J. Biol. Chem..

[50] Ma K., Xiao J., Li X., Zhang Q., Lian X. (2009). Sequence and expression analysis of the C3HC4-type RING finger gene family in rice. Gene.

[51] Cabral A.L., Jordan M.C., Larson G., Somers D.J., Humphreys D.G., McCartney C.A. (2018). Relationship between QTL for grain shape, grain weight, test weight, milling yield, and plant height in the spring wheat cross RL4452/‘AC Domain’. PLoS ONE.

[52] Zhou J., Wang X., Jiao Y., Qin Y., Liu X., He K., Chen C., Ma L., Wang J., Xiong L. (2007). Global genome expression analysis of rice in response to drought and high-salinity stresses in shoot, flag leaf, and panicle. Plant Mol. Biol..

[53] Sourdille P., Cadalen T., Gay G., Gill B., Bernard M. (2002). Molecular and physical mapping of genes affecting awning in wheat. Plant Breed..

[54] Echeverry-Solarte M., Kumar A., Kianian S., Mantovani E.E., McClean P.E., Deckard E.L., Elias E., Simsek S., Alamri M.S., Hegstad J. (2015). Genome-wide mapping of spike-related and agronomic traits in a common wheat population derived from a supernumerary spikelet parent and an elite parent. Plant Genome.

[55] Li H.Y., Wang T.Y., Shi Y.S., Fu J.J., Song Y.C., Wang G.Y., Li Y. (2007). Isolation and characterization of induced genes under drought stress at the flowering stage in maize (*Zea mays*): Full Length Research Paper. DNA Seq.

[56] Pandey S., Assmann S.M. (2004). The Arabidopsis putative g protein–coupled receptor GCR1 interacts with the G protein α subunit GPA1 and regulates abscisic acid signaling. Plant Cell.

[57] Wu Q., Chen Y., Fu L., Zhou S., Chen J., Zhao X., Zhang D., Ouyang S., Wang Z., Li D. (2016). QTL mapping of flag leaf traits in common wheat using an integrated high-density SSR and SNP genetic linkage map. Euphytica.

[58] Yang D., Liu Y., Cheng H., Chang L., Chen J., Chai S., Li M. (2016). Genetic dissection of flag leaf morphology in wheat (*Triticum aestivum* L.) under diverse water regimes. BMC Genet.

[59] Fan X., Cui F., Zhao C., Zhang W., Yang L., Zhao X., Han J., Su Q., Ji J., Zhao Z. (2015). QTLs for flag leaf size and their influence on yield-related traits in wheat (*Triticum aestivum* L.). Mol. Breed..

[60] Liu K., Xu H., Liu G., Guan P., Zhou X., Peng H., Yao Y., Ni Z., Sun Q., Du J. (2018). QTL mapping of flag leaf-related traits in wheat (*Triticum aestivum* L.). Theor. Appl. Genet..

[61] Hussain W., Baenziger P.S., Belamkar V., Guttieri M.J., Venegas J.P., Easterly A., Sallam A., Poland J. (2017). Genotyping-by-sequencing derived high-density linkage map and its application to QTL mapping of flag leaf traits in bread wheat. Sci. Rep..

[62] Ros B., Thümmler F., Wenzel G. (2004). Analysis of differentially expressed genes in a susceptible and moderately resistant potato cultivar upon Phytophthora infestans infection. Mol. Plant Pathol..

[63] Zhao Y., Cai M., Zhang X., Li Y., Zhang J., Zhao H., Kong F., Zheng Y., Qiu F. (2014). Genome-Wide identification, evolution and expression analysis of mTERF gene family in maize. PLoS ONE.

[64] Cotsaftis O., Plett D., Johnson A.A.T., Walia H., Wilson C., Ismail A.M., Close T.J., Tester M., Baumann U. (2011). Root-specific transcript profiling of contrasting rice genotypes in response to salinity stress. Mol. Plant.

[65] Hai L., Guo H., Xiao S., Jiang G., Zhang X., Yan C., Xin Z., Jia J. (2005). Quantitative trait loci (QTL) of stem strength and related traits in a doubled-haploid population of wheat (*Triticum aestivum* L.). Euphytica.

[66] Shumayla, Sharma S., Kumar R., Mendu V., Singh K., Upadhyay S.K. (2016). Genomic dissection and expression profiling revealed functional divergence in Triticum aestivum leucine rich repeat receptor like kinases (TaLRRKs). Front. Plant Sci..

[67] Landjeva S., Neumann K., Lohwasser U., Börner A. (2008). Molecular mapping of genomic regions associated with wheat seedling growth under osmotic stress. Biol. Plant..

[68] Ren Y., He X., Liu D., Li J., Zhao X., Li B., Tong Y., Zhang A., Li Z. (2012). Major quantitative trait loci for seminal root morphology of wheat seedlings. Mol. Breed..

[69] Bai C., Liang Y., Hawkesford M.J. (2013). Identification of QTLs associated with seedling root traits and their correlation with plant height in wheat. J. Exp. Bot..

[70] Atkinson J.A., Wingen L.U., Griffiths M., Pound M.P., Gaju O., Foulkes M.J., Le Gouis J., Griffiths S., Bennett M.J., King J. (2015). Phenotyping pipeline reveals major seedling root growth QTL in hexaploid wheat. J. Exp. Bot..

[71] Canè M.A., Maccaferri M., Nazemi G., Salvi S., Francia R., Colalongo C., Tuberosa R. (2014). Association mapping for root architectural traits in durum wheat seedlings as related to agronomic performance. Mol. Breed..

[72] Maccaferri M., El-Feki W., Nazemi G., Salvi S., Canè M.A., Colalongo M.C., Stefanelli S., Tuberosa R. (2016). Prioritizing quantitative trait loci for root system architecture in tetraploid wheat. J. Exp. Bot..

[73] Oono Y., Yazawa T., Kanamori H., Sasaki H., Mori S., Handa H., Matsumoto T. (2016). Genome-wide transcriptome analysis of cadmium stress in rice. BioMed Res. Int..

[74] Furukawa J., Yamaji N., Wang H., Mitani N., Murata Y., Sato K., Katsuhara M., Takeda K., Ma J.F. (2007). An aluminum-activated citrate transporter in barley. Plant Cell Physiol..

[75] Ryan P.R., Raman H., Gupta S., Horst W.J., Delhaize E. (2009). A second mechanism for aluminum resistance in wheat relies on the constitutive efflux of citrate from roots. Plant Physiol..

[76] Baron K.N., Schroeder D.F., Stasolla C. (2014). GEm-related 5 (GER5), an ABA and stress-responsive GRAM domain protein regulating seed development and inflorescence architecture. Plant Sci..

[77] Liu L., Li N., Yao C., Meng S., Song C. (2013). Functional analysis of the ABA-responsive protein family in ABA and stress signal transduction in *Arabidopsis*. Chin. Sci. Bull..

[78] Liu X., Huang M., Fan B., Buckler E.S., Zhang Z. (2016). Iterative usage of fixed and random effect models for powerful and efficient genome-wide association studies. PLoS Genet..

[79] Arora S., Singh N., Kaur S., Bains N.S., Uauy C., Poland J., Chhuneja P. (2017). Genome-wide association study of grain architecture in wild wheat Aegilops tauschii. Front. Plant Sci..

[80] Sukumaran S., Xiang W., Bean S.R., Pedersen J.F., Kresovich S., Tuinstra M.R., Tesso T.T., Hamblin M.T., Yu J. (2012). Association mapping for grain quality in a diverse sorghum collection. Plant Genome J..

[81] Barrett J.C., Fry B., Maller J., Daly M.J. (2005). Haploview: analysis and visualization of LD and haplotype maps. Bioinformatics.

[82] Singh A., Giri J., Kapoor S., Tyagi A.K., Pandey G.K. (2010). Protein phosphatase complement in rice: Genome-wide identification and transcriptional analysis under abiotic stress conditions and reproductive development. BMC Genom..

[83] (2013). Evaluation of deficit irrigation for efficient sheep production from permanent sown pastures in a dry continental climate. Agric. Water Manag..

[84] SAS 9.4 Product Documentation. https://support.sas.com/documentation/94/.

[85] Kassambara A., Mundt F. (2017). Factoextra: Extract and visualize the results of multivariate data analyses. http://www.sthda.com/english/rpkgs/factoextra/.

[86] Poland J.A., Brown P.J., Sorrells M.E., Jannink J. (2012). Development of high-density genetic maps for barley and wheat using a novel two-enzyme genotyping-by-sequencing approach. PLoS ONE.

[87] Glaubitz J.C., Casstevens T.M., Lu F., Harriman J., Elshire R.J., Sun Q., Buckler E.S. (2014). TASSEL-GBS: A high capacity genotyping by sequencing analysis pipeline. PLoS ONE.

[88] International Wheat Genome Sequencing Consortium (IWGSC) (2018). Shifting the limits in wheat research and breeding using a fully annotated reference genome. Science.

[89] Pritchard J.K., Stephens M., Donnelly P. (2000). Inference of Population Structure Using Multilocus Genotype Data. Genetics.

[90] Bradbury P.J., Zhang Z., Kroon D.E., Casstevens T.M., Ramdoss Y., Buckler E.S. (2007). TASSEL: Software for association mapping of complex traits in diverse samples. Bioinformatics.

[91] Yu J., Pressoir G., Briggs W.H., Vroh Bi I., Yamasaki M., Doebley J.F., McMullen M.D., Gaut B.S., Nielsen D.M., Holland J.B. (2006). A unified mixed-model method for association mapping that accounts for multiple levels of relatedness. Nat. Genet..

[92] Zhang Z., Ersoz E., Lai C.-Q., Todhunter R.J., Tiwari H.K., Gore M.A., Bradbury P.J., Yu J., Arnett D.K., Ordovas J.M. (2010). Mixed linear model approach adapted for genome-wide association studies. Nature Genetics.

[93] Cingolani P., Platts A., Wang L.L., Coon M., Nguyen T., Wang L., Land S.J., Lu X., Ruden D.M. (2012). A program for annotating and predicting the effects of single nucleotide polymorphisms, SnpEff. Fly (Austin).

