# Supplementary material for: Genome-Wide Association Study Reveals Novel Genomic Regions for Grain Yield and Yield-Related Traits in Drought-Stressed Synthetic Hexaploid Wheat
Source: Int J Mol Sci. 2018 Oct 2;19(10):3011. doi: 10.3390/ijms19103011 (PMC6212811; doi:10.3390/ijms19103011)
Supplement: Supplementary file 1 [file ijms-19-03011-s001.zip › ijms-352624 Supplementary for proof/Figure S1.pdf]

Figure S1. Pearson's correlation of grain yield and related traits of 123 synthetic hexaploid wheat based on adjusted best linear unbiased predictors in 2016 (A) and 2017 (B) growing season in Konya, Turkey.

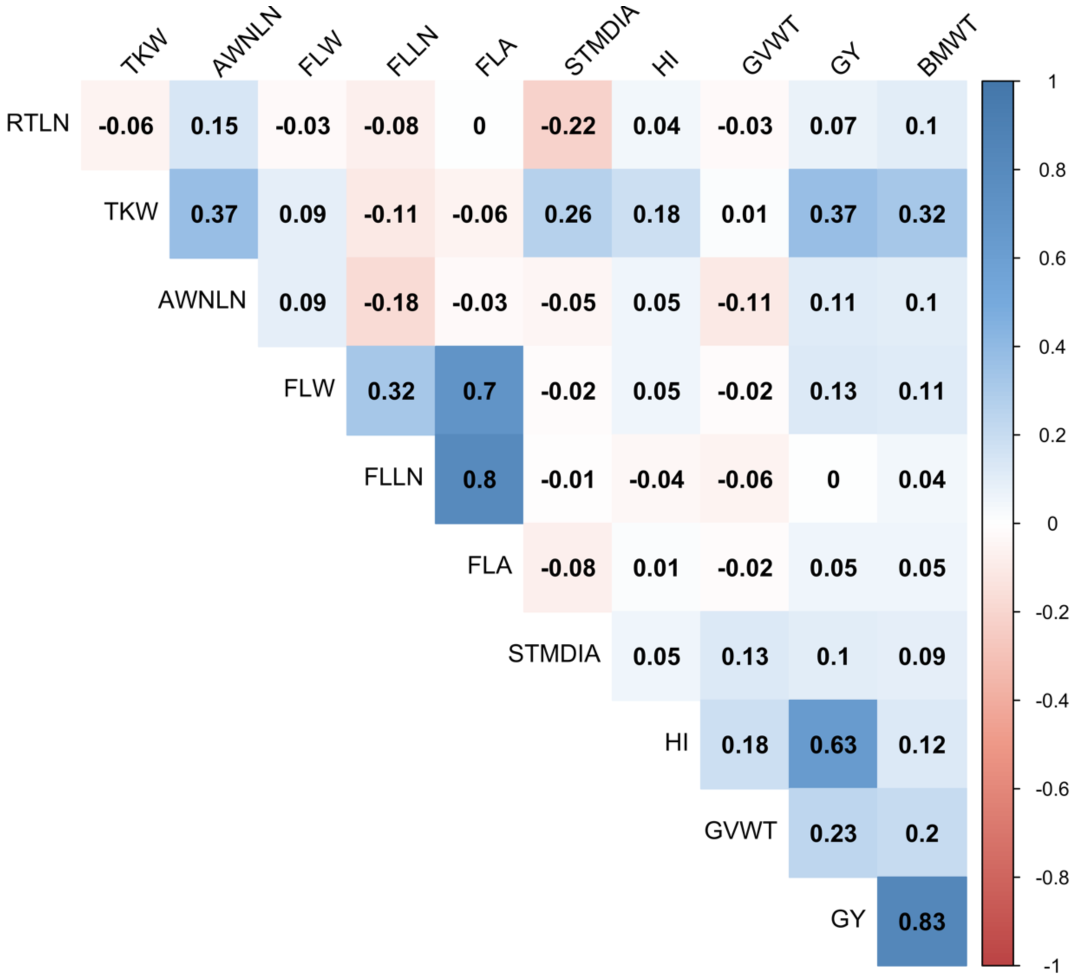

A. Correlation plot for grain yield and yield-related traits in 2016 experiment.

GY, Grain yield; HI, Harvest index; BMWT, Biomass weight; TKW, Thousand kernel weight, GVWT, Grain volume weight; AWNLN, Awn length; FLLN, Flag leaf length; FLW, Flag leaf width; FLA, Flag leaf area; STMDIA, Stem diameter; RTLN, Root length

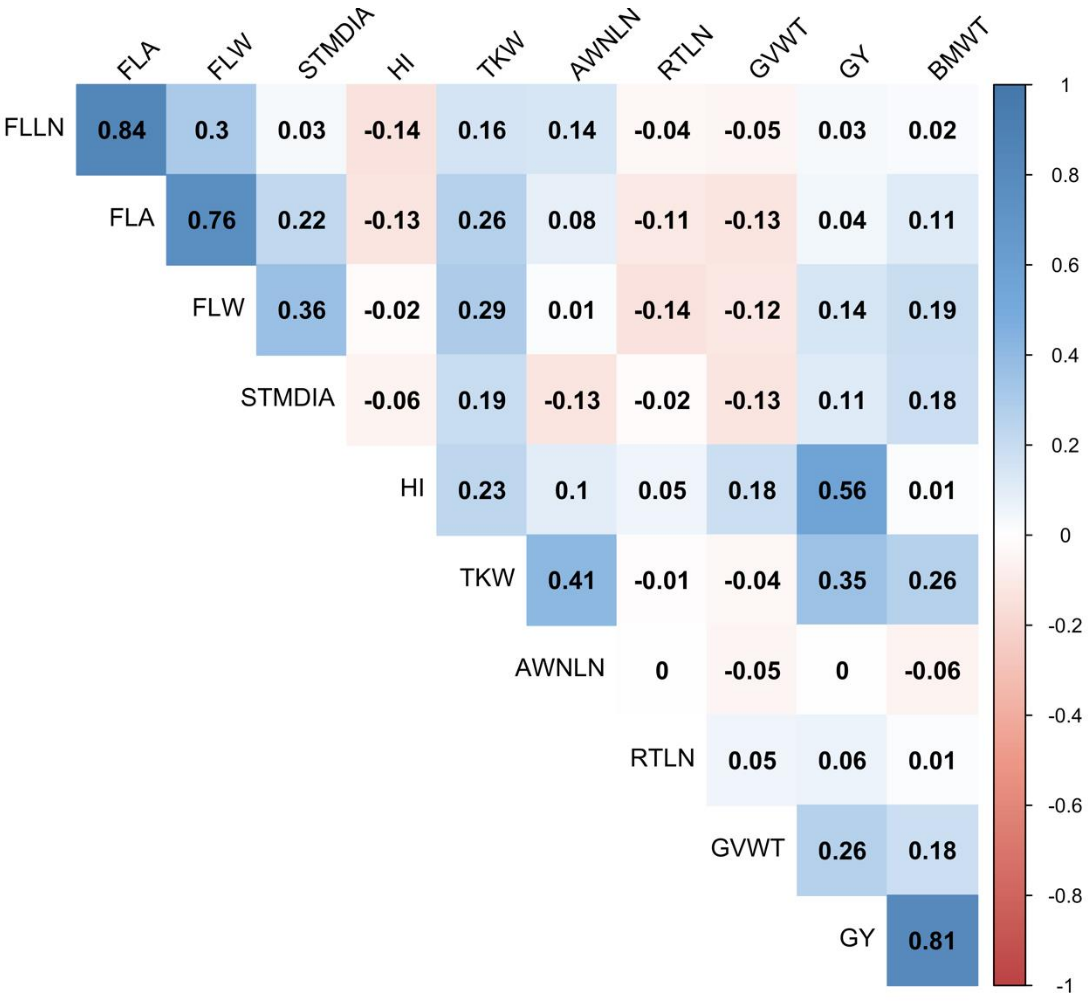

B. Correlation plot for grain yield and yield-related traits in 2017 experiment.

GY, Grain yield; HI, Harvest index; BMWT, Biomass weight; TKW, Thousand kernel weight, GVWT, Grain volume weight; AWNLN, Awn length; FLLN, Flag leaf length; FLW, Flag leaf width; FLA, Flag leaf area; STMDIA, Stem diameter; RTLN, Root length.
