# Supplementary material for: Genome-Wide Association Study Reveals Novel Genomic Regions for Grain Yield and Yield-Related Traits in Drought-Stressed Synthetic Hexaploid Wheat
Source: Int J Mol Sci. 2018 Oct 2;19(10):3011. doi: 10.3390/ijms19103011 (PMC6212811; doi:10.3390/ijms19103011)
Supplement: Supplementary file 1 [file ijms-19-03011-s001.zip › ijms-352624 Supplementary for proof/Figure S2.pdf]

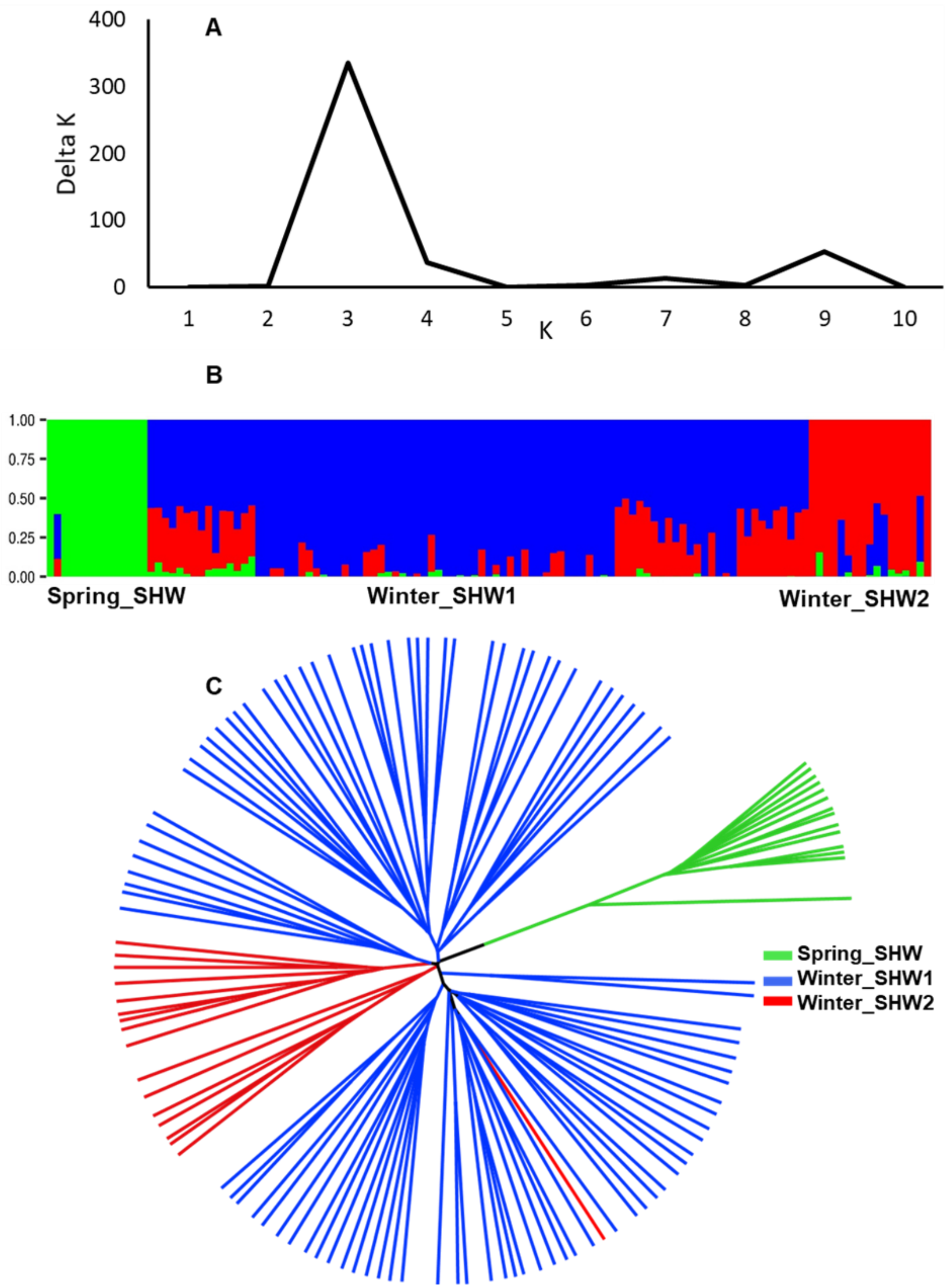

Figure S2. Population structure analysis of 123 synthetic hexaploid wheat. (**A**) Line graph of deltak K over K from 1 to 10, and the highest peak was observed delta K=3, suggesting SHW has three subgroups. (**B**) Three subgroups (Spring_SHW, Winter_SHW1, and Winter_SHW2) were identified from the STRUCTURE. (**C**) UPGMA cluster analysis.
