# Supplementary material for: Genome-Wide Association Study Reveals Novel Genomic Regions for Grain Yield and Yield-Related Traits in Drought-Stressed Synthetic Hexaploid Wheat
Source: Int J Mol Sci. 2018 Oct 2;19(10):3011. doi: 10.3390/ijms19103011 (PMC6212811; doi:10.3390/ijms19103011)
Supplement: Supplementary file 1 [file ijms-19-03011-s001.zip › ijms-352624 Supplementary for proof/Figure S3.pdf]

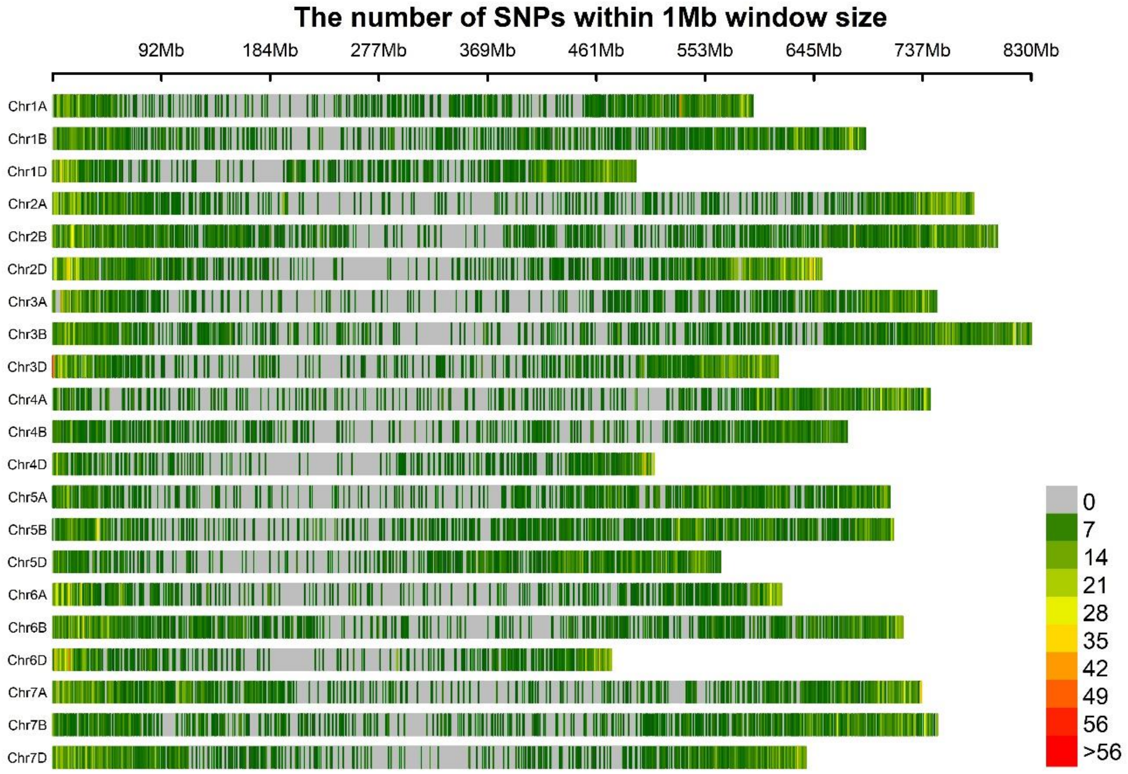

Figure S3. Physical distribution of genotyping-by-sequencing-derived SNPs within a 1 Mb window size on 21 chromosomes of 123 synthetic hexaploid wheat.
