# Supplementary material for: Genome-Wide Association Study Reveals Novel Genomic Regions for Grain Yield and Yield-Related Traits in Drought-Stressed Synthetic Hexaploid Wheat
Source: Int J Mol Sci. 2018 Oct 2;19(10):3011. doi: 10.3390/ijms19103011 (PMC6212811; doi:10.3390/ijms19103011)
Supplement: Supplementary file 1 [file ijms-19-03011-s001.zip › ijms-352624 Supplementary for proof/Figure S4.pdf]

Figure S4. Manhattan and quantile-quantile plots for 11 traits in 123 synthetic hexaploid wheat germplasm based on best linear unbiased prediction values.

(A) Manhattan plot in 2016 (B) quantile-quantile (Q-Q) plots in 2016 (C) Manhattan plot in 2017 (D) Q-Q plot in 2017

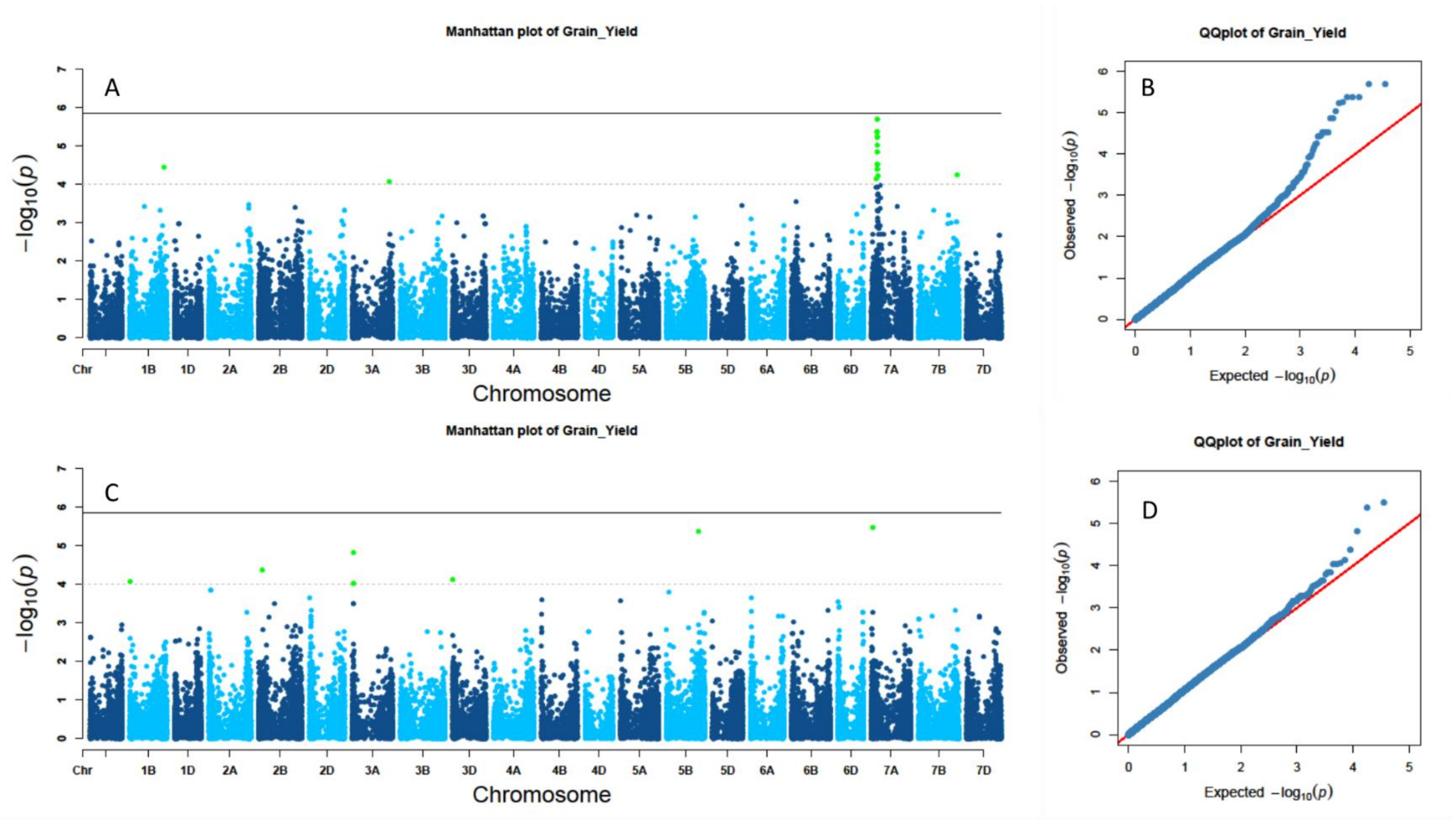

Grain yield (GY)

(A) Manhattan plot in 2016. (B) quantile-quantile (Q-Q) plots in 2016. (C) Manhattan plot in 2017. (D) Q-Q plot in 2017. The solid black line showed the expected value at Bonferroni correction at 5% level of significance [-$\log_{10}$(P) = 5.85] and dotted black line showed the expected value with a uniform suggestive genome wide significance threshold [-$\log_{10}$(P) = 4.00].

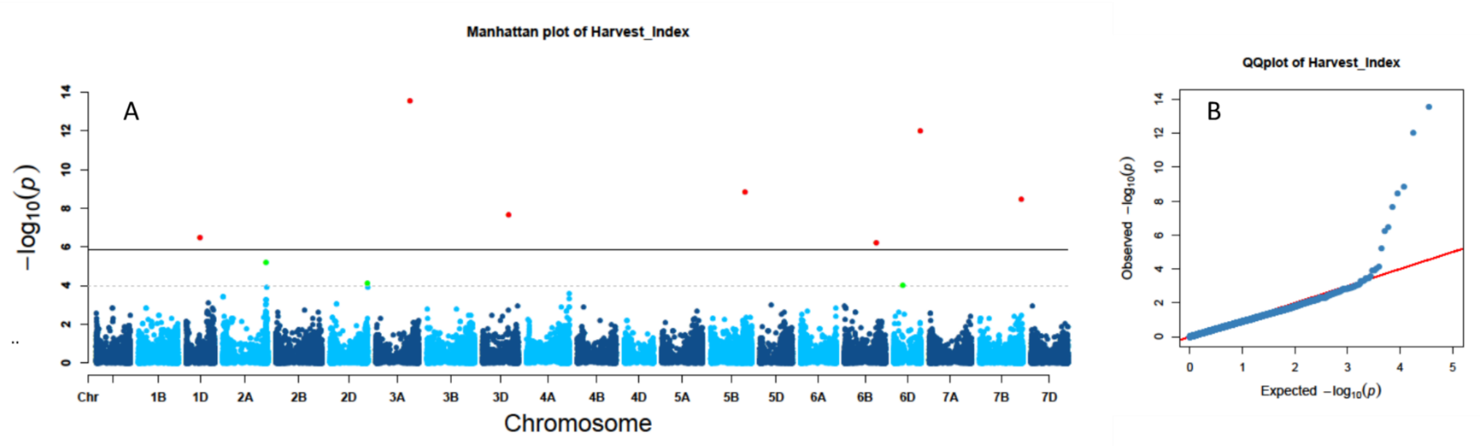

Harvest index (HI)

(A) Manhattan plot in 2016. (B) quantile-quantile (Q-Q) plots in 2016. The solid black line showed the expected value at Bonferroni correction at 5% level of significance [$-\log_{10}(P) = 5.85$] and dotted black line showed the expected value with a uniform suggestive genome wide significance threshold [$-\log_{10}(P) = 4.00$].

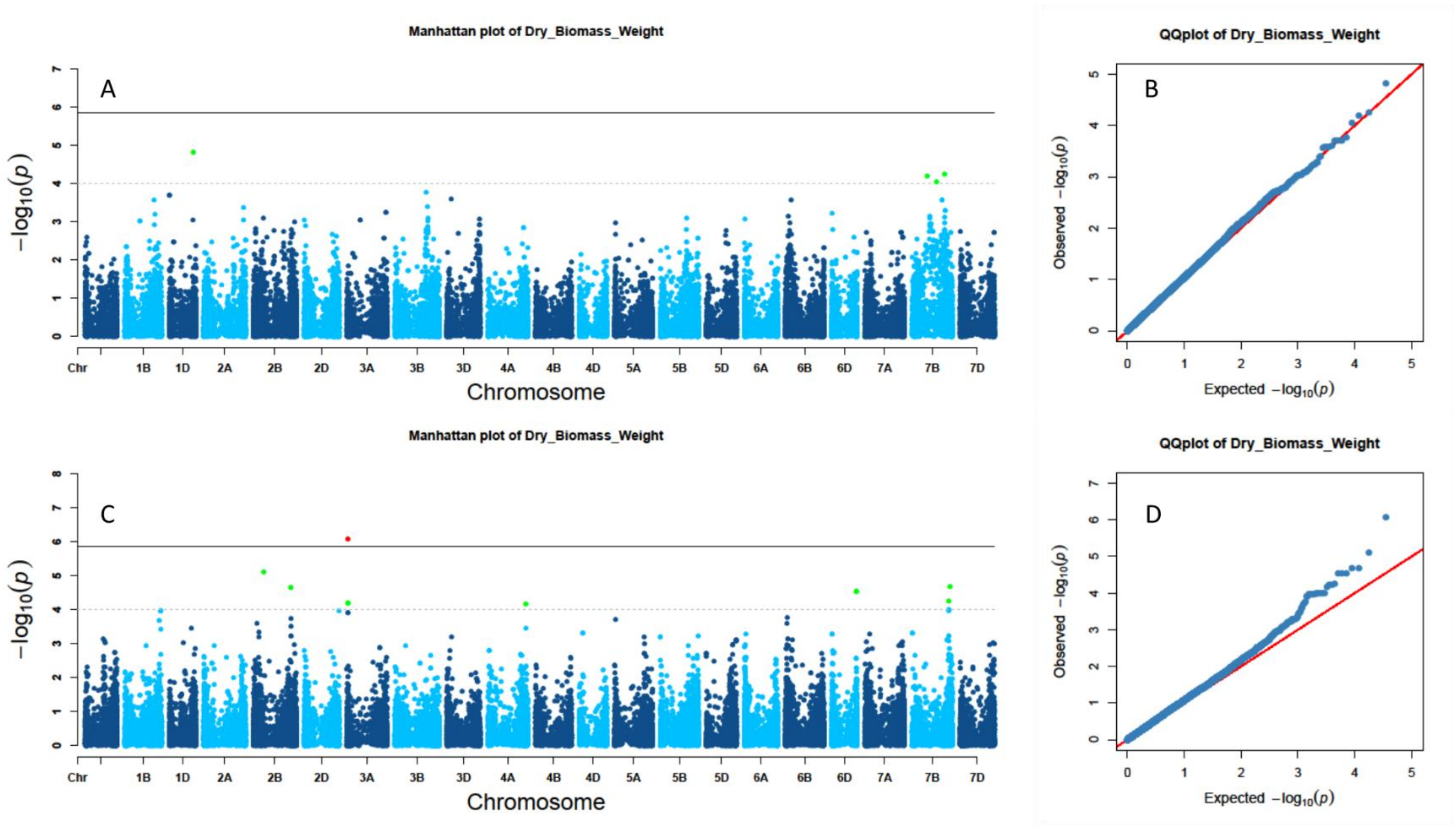

Dry biomass weight (BMWT)

(A) Manhattan plot in 2016. (B) quantile-quantile (Q-Q) plots in 2016. (C) Manhattan plot in 2017. (D) Q-Q plot in 2017. The solid black line showed the expected value at Bonferroni correction at 5% level of significance [$-\log_{10}(P) = 5.85$] and dotted black line showed the expected value with a uniform suggestive genome wide significance threshold [$-\log_{10}(P) = 4.00$].

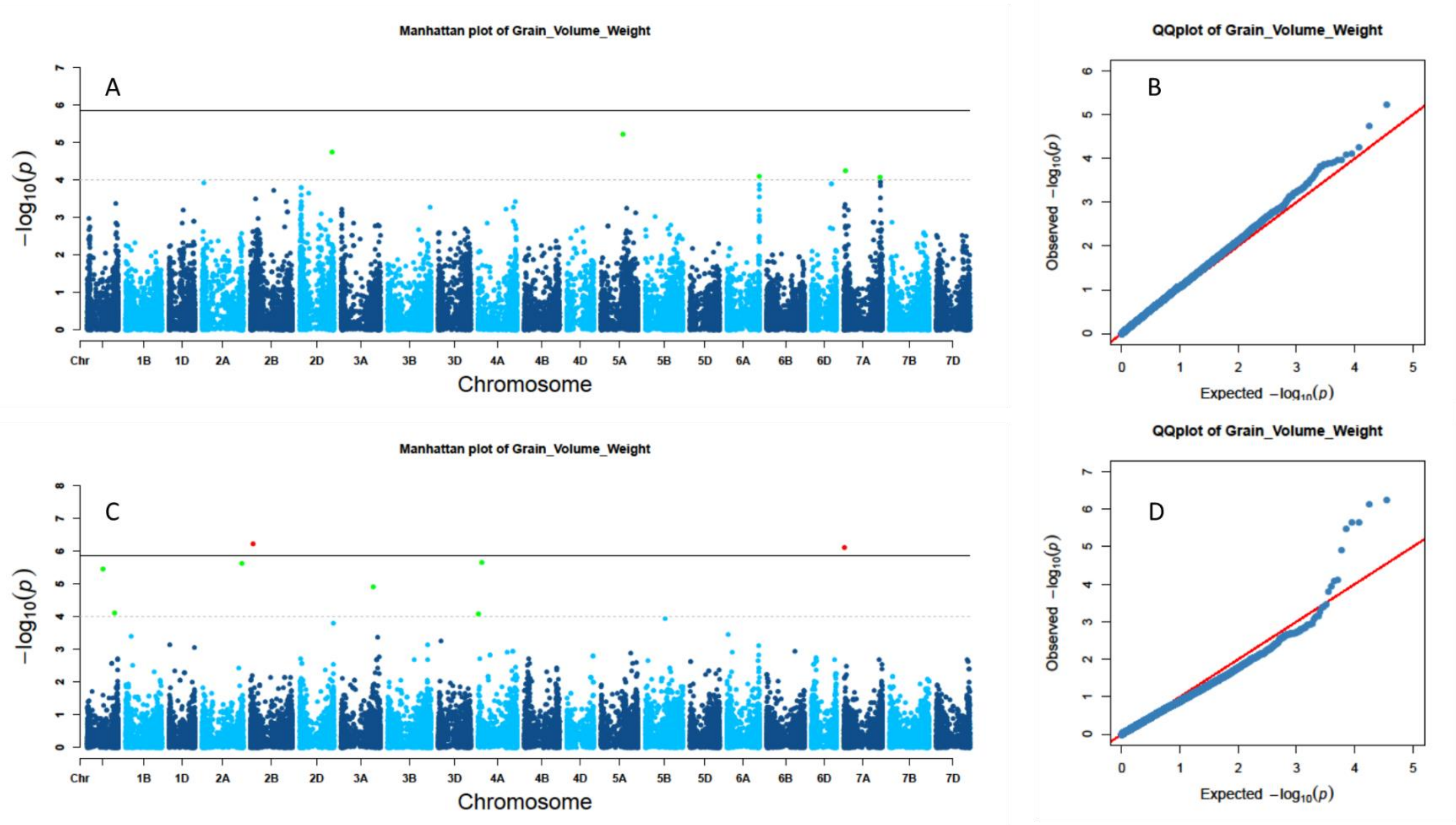

Grain volume weight (GVWT)

(A) Manhattan plot in 2016. (B) quantile-quantile (Q-Q) plots in 2016. (C) Manhattan plot in 2017. (D) Q-Q plot in 2017. The solid black line showed the expected value at Bonferroni correction at 5% level of significance [$-\log_{10}(P) = 5.85$] and dotted black line showed the expected value with a uniform suggestive genome wide significance threshold [$-\log_{10}(P) = 4.00$].

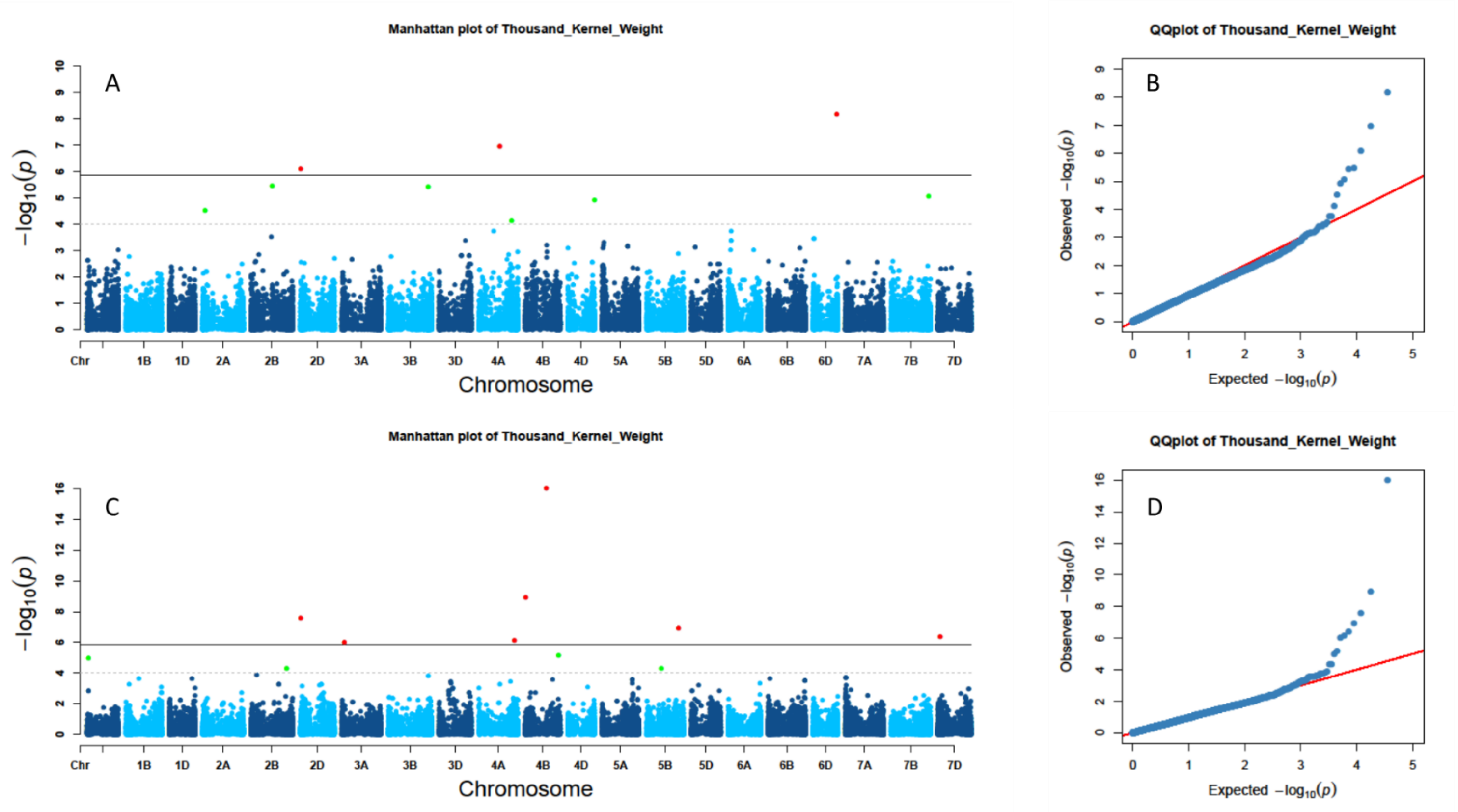

Thousand kernel weight (TKW)

(A) Manhattan plot in 2016. (B) quantile-quantile (Q-Q) plots in 2016. (C) Manhattan plot in 2017. (D) Q-Q plot in 2017. The solid black line showed the expected value at Bonferroni correction at 5% level of significance [$-\log_{10}(P) = 5.85$] and dotted black line showed the expected value with a uniform suggestive genome wide significance threshold [$-\log_{10}(P) = 4.00$].

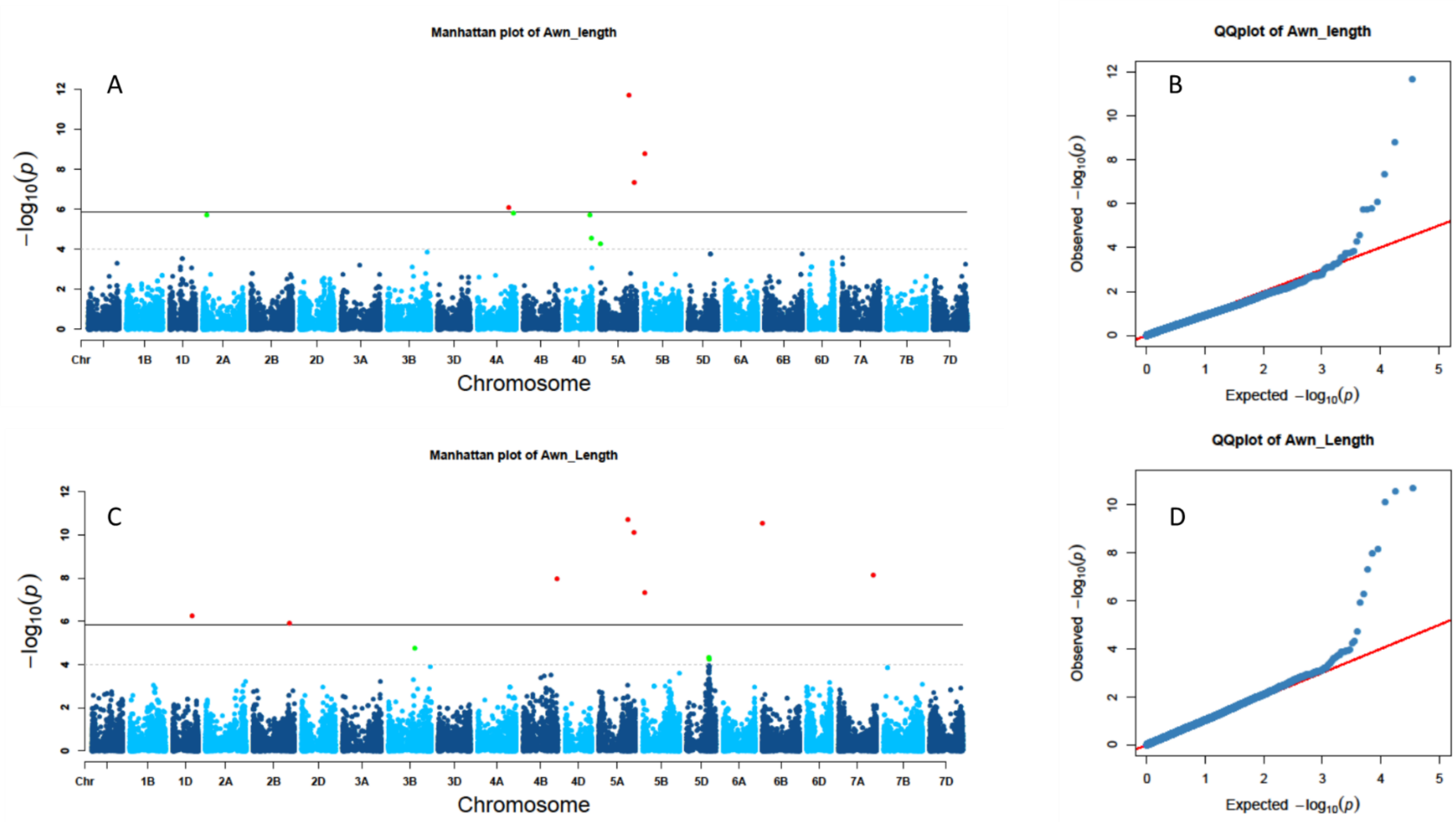

Awn length (AWNLN)

(A) Manhattan plot in 2016. (B) quantile-quantile (Q-Q) plots in 2016. (C) Manhattan plot in 2017. (D) Q-Q plot in 2017. The solid black line showed the expected value at Bonferroni correction at 5% level of significance [$-\log_{10}(P) = 5.85$] and dotted black line showed the expected value with a uniform suggestive genome wide significance threshold [$-\log_{10}(P) = 4.00$].

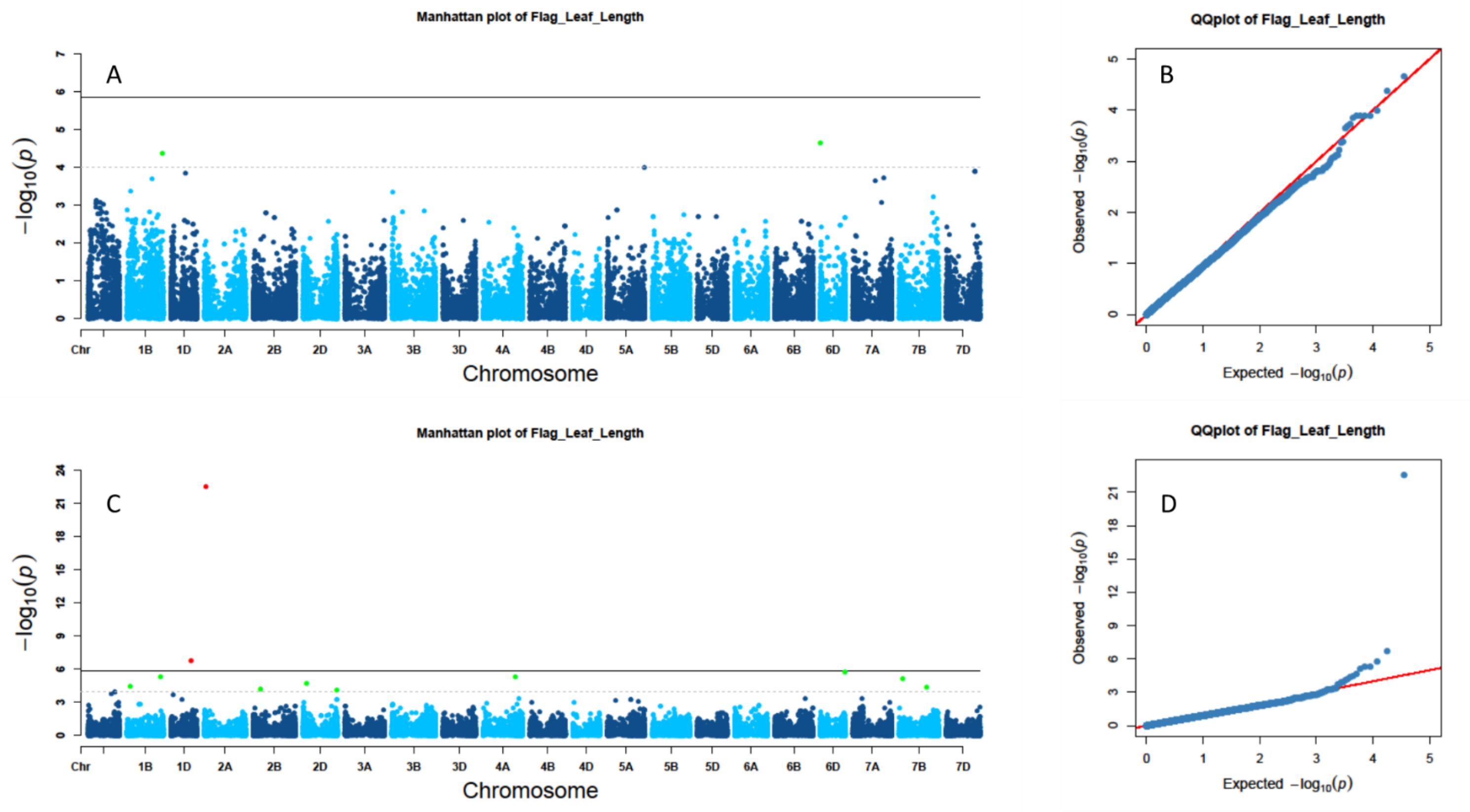

Flag leaf length (FLLN)

(A) Manhattan plot in 2016. (B) quantile-quantile (Q-Q) plots in 2016. (C) Manhattan plot in 2017. (D) Q-Q plot in 2017. The solid black line showed the expected value at Bonferroni correction at 5% level of significance [$-\log_{10}(P) = 5.85$] and dotted black line showed the expected value with a uniform suggestive genome wide significance threshold [$-\log_{10}(P) = 4.00$].

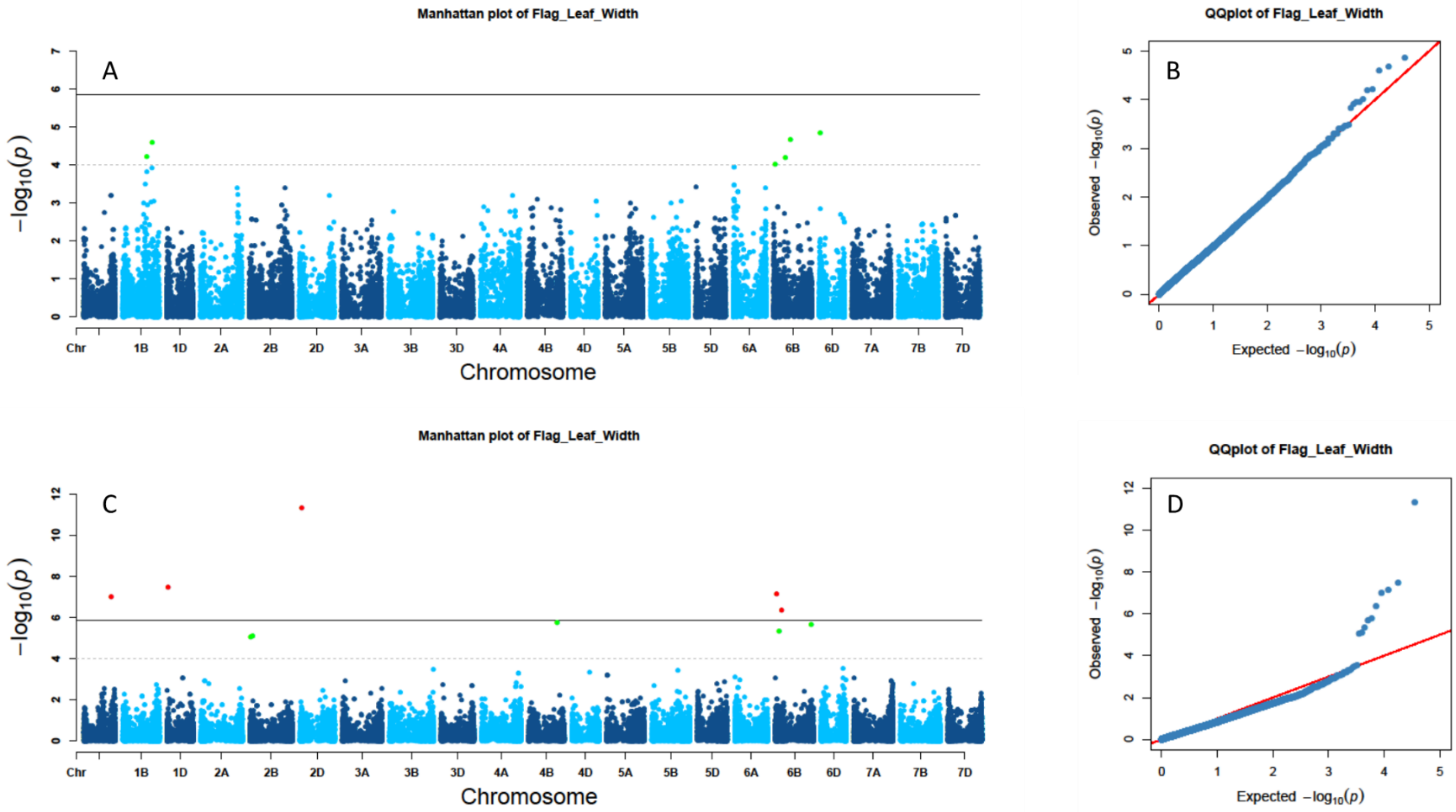

Flag leaf width (FLW)

(A) Manhattan plot in 2016. (B) quantile-quantile (Q-Q) plots in 2016. (C) Manhattan plot in 2017. (D) Q-Q plot in 2017. The solid black line showed the expected value at Bonferroni correction at 5% level of significance [$-\log_{10}(P) = 5.85$] and dotted black line showed the expected value with a uniform suggestive genome wide significance threshold [$-\log_{10}(P) = 4.00$].

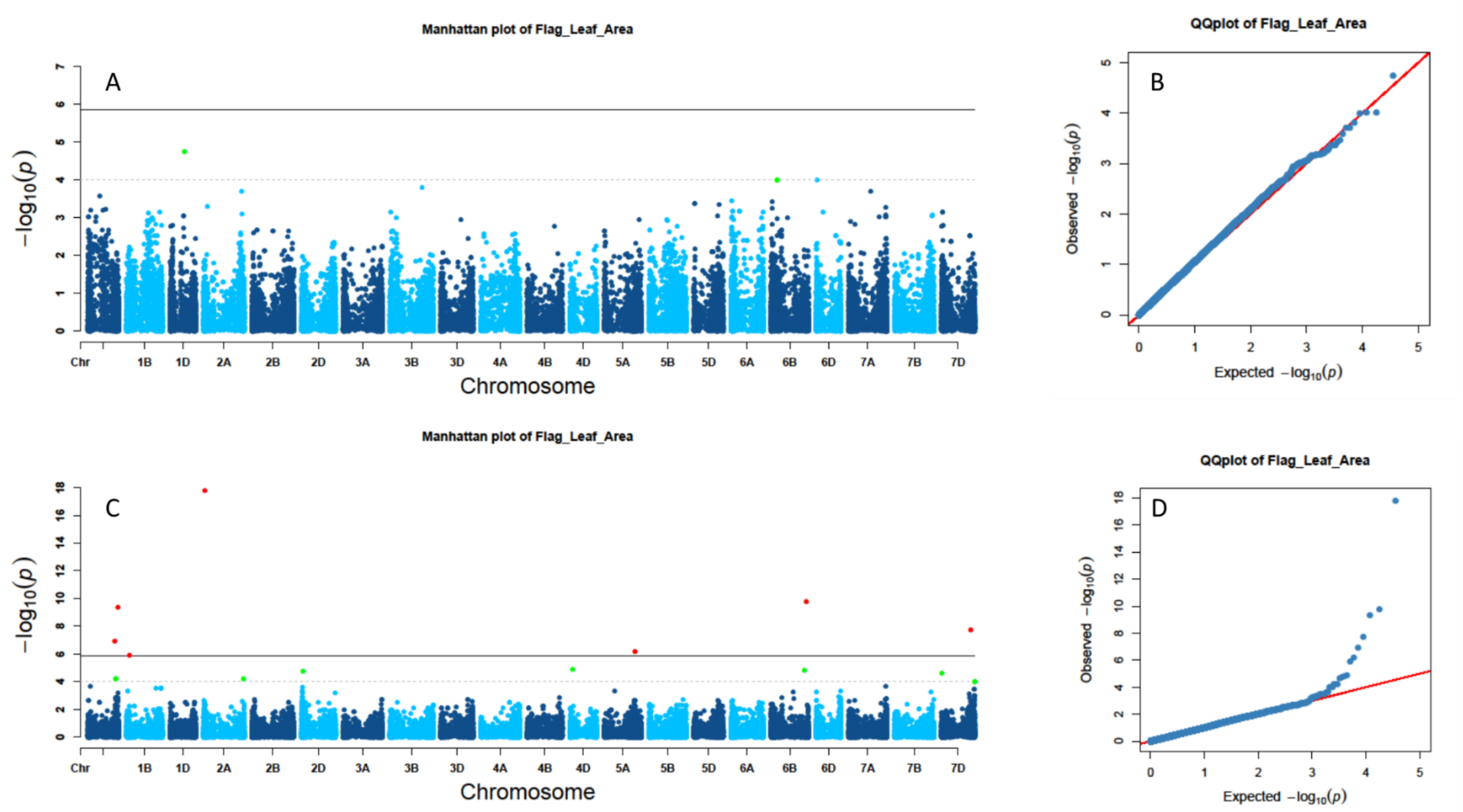

Flag leaf area (FLA)

(A) Manhattan plot in 2016. (B) quantile-quantile (Q-Q) plots in 2016. (C) Manhattan plot in 2017. (D) Q-Q plot in 2017. The solid black line showed the expected value at Bonferroni correction at 5% level of significance [$-\log_{10}(P) = 5.85$] and dotted black line showed the expected value with a uniform suggestive genome wide significance threshold [$-\log_{10}(P) = 4.00$].

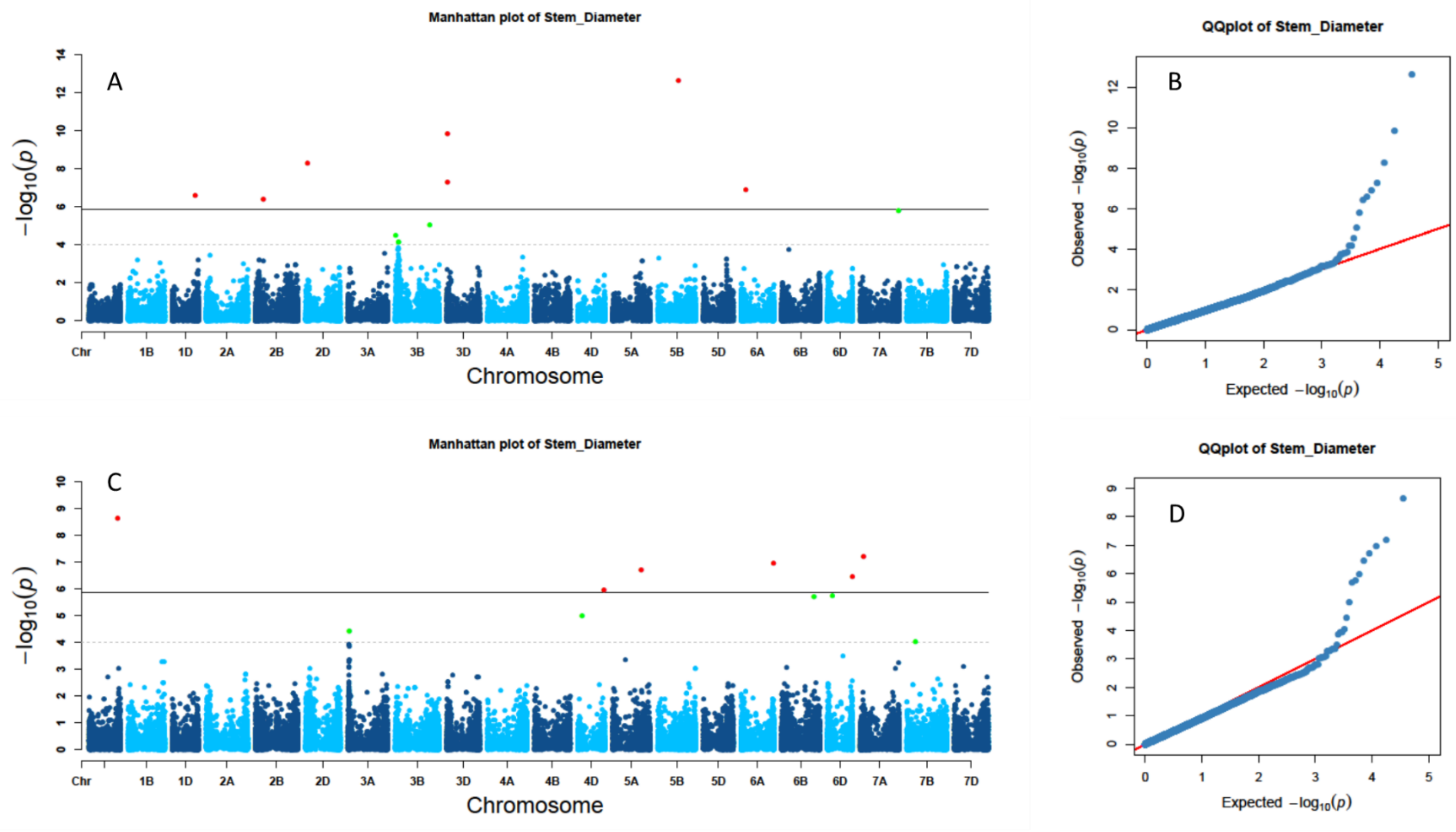

Stem diameter (STMDIA)

(A) Manhattan plot in 2016. (B) quantile-quantile (Q-Q) plots in 2016. (C) Manhattan plot in 2017. (D) Q-Q plot in 2017. The solid black line showed the expected value at Bonferroni correction at 5% level of significance [$-\log_{10}(P) = 5.85$] and dotted black line showed the expected value with a uniform suggestive genome wide significance threshold [$-\log_{10}(P) = 4.00$].

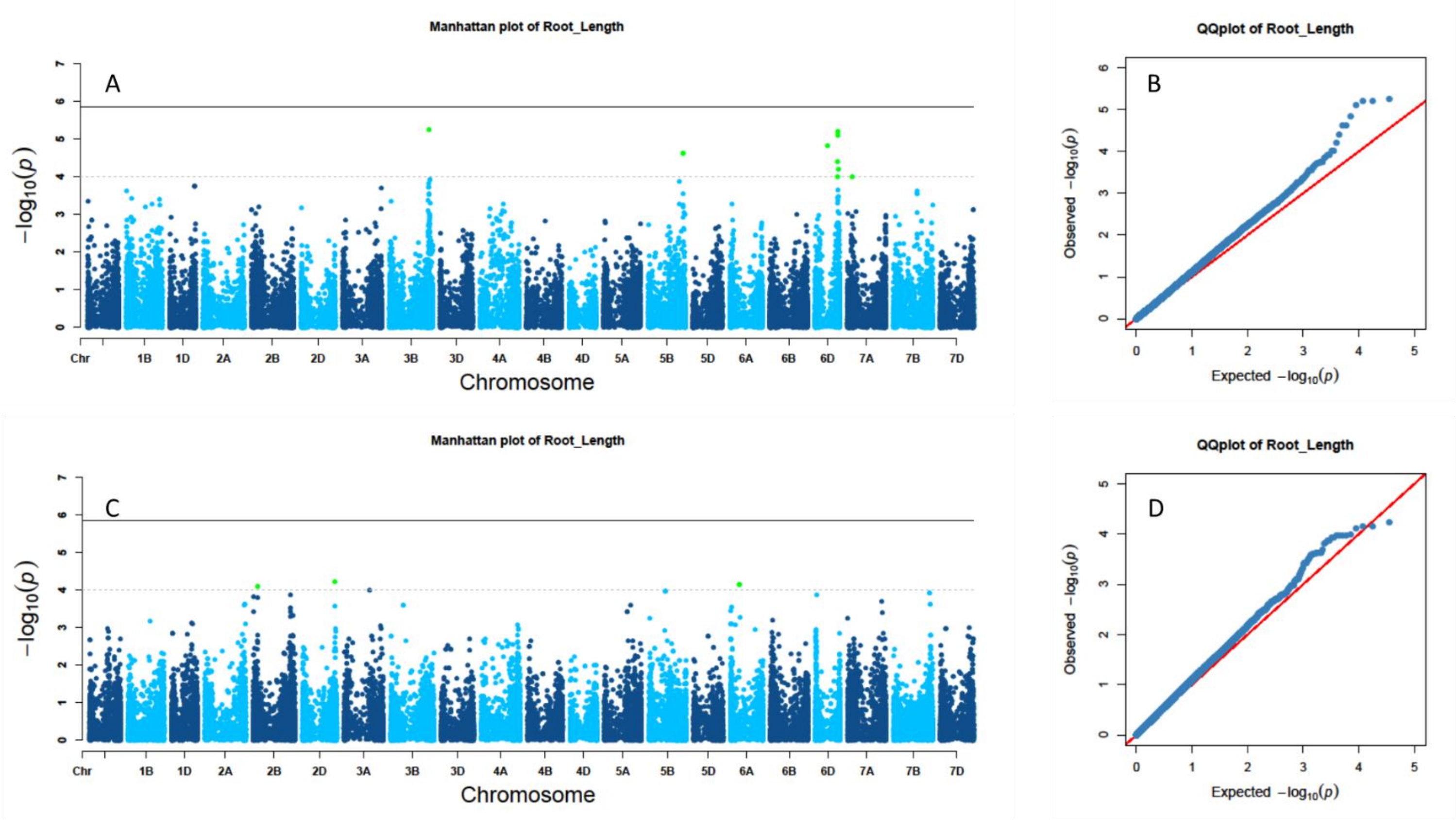

Root length (RTLN)

(A) Manhattan plot in 2016. (B) quantile-quantile (Q-Q) plots in 2016. (C) Manhattan plot in 2017. (D) Q-Q plot in 2017. The solid black line showed the expected value at Bonferroni correction at 5% level of significance [$-\log_{10}(P) = 5.85$] and dotted black line showed the expected value with a uniform suggestive genome wide significance threshold [$-\log_{10}(P) = 4.00$].
