# Supplementary material for: Genome-Wide Association Study Reveals Novel Genomic Regions for Grain Yield and Yield-Related Traits in Drought-Stressed Synthetic Hexaploid Wheat
Source: Int J Mol Sci. 2018 Oct 2;19(10):3011. doi: 10.3390/ijms19103011 (PMC6212811; doi:10.3390/ijms19103011)
Supplement: Supplementary file 1 [file ijms-19-03011-s001.zip › ijms-352624 Supplementary for proof/Public database link.pdf]

# Genome-Wide Association Study Reveals Novel Genomic Regions for Grain Yield and Yield-Related Traits in Drought-Stressed Synthetic Hexaploid Wheat

**All supplementary materials are available online:** https://figshare.com/s/c448a2d5a559b3f6b462

**Figure S1.** Pearson's correlation of grain yield and yield-related traits of 123 synthetic hexaploid wheat based on adjusted best linear unbiased predictors in 2016 (A) and 2017 (B) growing seasons in Konya, Turkey.

**Figure S2.** Population structure analysis of 123 synthetic hexaploid wheat.

**Figure S3.** Physical distribution of genotyping-by-sequencing-derived SNPs within a 1 Mb window size on 21 chromosomes of 123 synthetic hexaploid wheat.

**Figure S4.** Manhattan and quantile-quantile plots for 11 traits in 123 synthetic hexaploid wheat germplasm.

**Table S1.** Details of 123 synthetic hexaploid wheat used and subgroups obtained from the Bayesian clustering algorithm with population structure matrix (Q) and BLUPs from two-year (2016 and 2017) experiments conducted in Konya, Turkey.

**Table S2.** Crossing scheme of 123 synthetic hexaploid wheat.

**Table S3.** Genotyping-by-sequencing-derived SNP markers of 123 synthetic hexaploid wheats used in the present study.

**Table S4.** Combined analysis of variance with means squares and broad sense heritability ($H^2$) of 123 drought stressed synthetic hexaploid wheats grown in two seasons (2016 and 2017) in Konya, Turkey.

**Table S5.** Details of significant markers associated with 11 traits in 123 synthetic hexaploid wheat grown in two seasons (2016 and 2017) in Konya, Turkey.

**Table S6.** Details of potential candidate gene functions harboring SNPs affecting yield and yield-related traits under drought stress in 123 synthetic hexaploid wheat grown in two seasons (2016 and 2017) in Konya, Turkey.
