# Supplementary material for: Genome-Wide Association Study Reveals Novel Genomic Regions for Grain Yield and Yield-Related Traits in Drought-Stressed Synthetic Hexaploid Wheat
Source: Int J Mol Sci. 2018 Oct 2;19(10):3011. doi: 10.3390/ijms19103011 (PMC6212811; doi:10.3390/ijms19103011)
Supplement: Supplementary file 1 [file ijms-19-03011-s001.zip › ijms-352624 Supplementary for proof/Table S4.pdf]

**Table S4**. Combined analysis of variance with means squares and broad sense heritability ($H^2$) of 123 drought stressed synthetic hexaploid wheat grown in two seasons (2016 and 2017) in Konya, Turkey.

| Source | DF | Grain yield | Harvest index | Biomass weight | Thousand kernel weight | Grain volume weight | Awn length | Flag leaf length | Flag leaf width | Flag leaf area | Stem diameter | Root length |
|---|---|---|---|---|---|---|---|---|---|---|---|---|
| Year (YR) | 1 | 9982 | 0.0073 | 229461 | 411*** | 84.3*** | 0.3 | 399*** | 0.0005 | 264*** | 0.04 | 263427*** |
| Replication(R) | 1 | 94696*** | 0.0101* | 467260** | 1 | 0 | 1.5 | 5 | 0.1486** | 33** | 0.06 | 7914 |
| iblock(R*YR) | 54 | 5181** | 0.0018* | 43534*** | 3 | 2.2 | 0.5 | 2 | 0.0147 | 4 | 0.11 | 3177 |
| Check | 1 | 100 | 0.0001 | 2190 | 12 | 4.4 | 2.9* | 1 | 0.3397*** | 52** | 0.33 | 15824 |
| Genotype(G) | 122 | 5967* | 0.0038* | 27184* | 27*** | 18.6** | 5.8*** | 8* | 0.0276*** | 14* | 0.2*** | 9790* |
| G X YR | 123 | 4376* | 0.0027*** | 20126* | 8*** | 10.9*** | 1.4*** | 7.7** | 0.0152 | 12** | 0.11 | 8510* |
| Residual | 87 | 2869 | 0.0011 | 13372 | 4 | 2.6 | 0.6 | 4 | 0.0161 | 6 | 0.08 | 6072 |
| $H^2$ | | 0.40 | 0.62 | 0.56 | 0.74 | 0.75 | 0.81 | 0.49 | 0.56 | 0.50 | 0.62 | 0.27 |

*, **, and *** Significant at the 0.05, 0.01, and 0.001 probability level, respectively.
